# Metabolomic profiles of bovine cumulus cells and cumulus-oocyte-complex-conditioned medium during maturation *in vitro*

**DOI:** 10.1038/s41598-018-27829-9

**Published:** 2018-06-21

**Authors:** Karen Uhde, Helena T. A. van Tol, Tom A. E. Stout, Bernard A. J. Roelen

**Affiliations:** 1 Department of Farm Animal Health, Faculty of Veterinary Medicine, Utrecht University, Utrecht, The Netherlands; 2 Department of Equine Sciences, Faculty of Veterinary Medicine, Utrecht University, Utrecht, The Netherlands

## Abstract

Cumulus cells are essential for nutrition of oocytes during maturation. In the absence of cumulus cells during maturation, oocyte developmental competence is severely compromised. In this study, we matured bovine cumulus-oocyte-complexes (COCs) for 8 h, the cumulus cells were removed and denuded oocytes were further matured for 15 h in either the medium conditioned by the initial 8 h culture, or in fresh unconditioned medium. Denuded oocytes that completed maturation in COC-conditioned medium demonstrated better developmental potential than denuded oocytes that completed maturation in standard maturation medium. An inventory was made of the metabolites secreted by COCs into the maturation medium during the first 8 h, from 8 to 23 h, and during an entire 23 h maturation protocol; the metabolomic changes in the cumulus cells during maturation were also investigated. In maturation medium, 173 biochemical components were detected compared to 369 different metabolites in cumulus cells. Significant changes in metabolomic components were evident in maturation medium and in cumulus cells during maturation, with most of the changes related to amino acid, carbohydrate, and lipid metabolism. The importance of two detected biochemicals, creatine and carnitine, for oocyte maturation was further investigated. The presence of carnitine, but not creatine during oocyte *in vitro* maturation improved the developmental competence of denuded oocytes.

## Introduction

The mammalian oocyte is surrounded by specialized somatic cells, cumulus cells, that support oocyte maturation and allow it to be fertilized and develop into a viable embryo. The final stage of oocyte maturation includes resumption of meiosis and storage of mRNA, proteins and nutrients required to support the early stages of embryo development before the embryonic genome is switched on. Cumulus cells are connected to the oocyte via transzonal cytoplasmic projections which enable the transfer of molecules between cumulus cells and the oocyte^1–5^. Maturation of oocytes in the absence of cumulus cells dramatically reduces the likelihood of fertilization and the competence to form an embryo, demonstrating the importance of the cumulus cells^6^. Nevertheless, during the first 3 hours of maturation there is already partial loss of transzonal cumulus cells processes^7–9^, indicating that communication through the cytoplasmic connections across the zona pellucida is particularly important during the early stages of oocyte maturation. Furthermore, culture of denuded oocytes in the presence of cumulus-oocyte-complexes (COCs) partially restores the developmental competence of oocytes^10^ indicating that, as well as gap junctional communication, factors secreted by the cumulus cells also positively influence oocyte development.

The exact components produced by cumulus cells that enhance the developmental competence of oocytes during maturation are, however, largely unknown. A better understanding of the functions of cumulus cells and cumulus cell products in the maturation of the enclosed oocyte may help to improve the success of *in vitro* maturation and fertilization.

To investigate the paracrine interactions between somatic cells and the oocyte, the developmental competence of bovine oocytes denuded 8 h after the onset of *in vitro* maturation (IVM) and thereafter matured in either fresh or COC-conditioned culture medium was examined. Oocytes were denuded 8 h after the start of maturation, because of the timing of the GVBD, which occurs between 6.6 and 8 h^11^. Additionally, the communication between the cumulus cells and the oocyte is completely lost after 9 h of maturation^12^.

In addition, the metabolic profiles of cumulus cells and COC-conditioned media were determined by mass spectrometry to identify candidate components or changes that may support the oocyte’s acquisition of developmental competence.

## Results

### COCs secrete maturation enhancing factors during the first 8h of maturation

**Figure 1:**
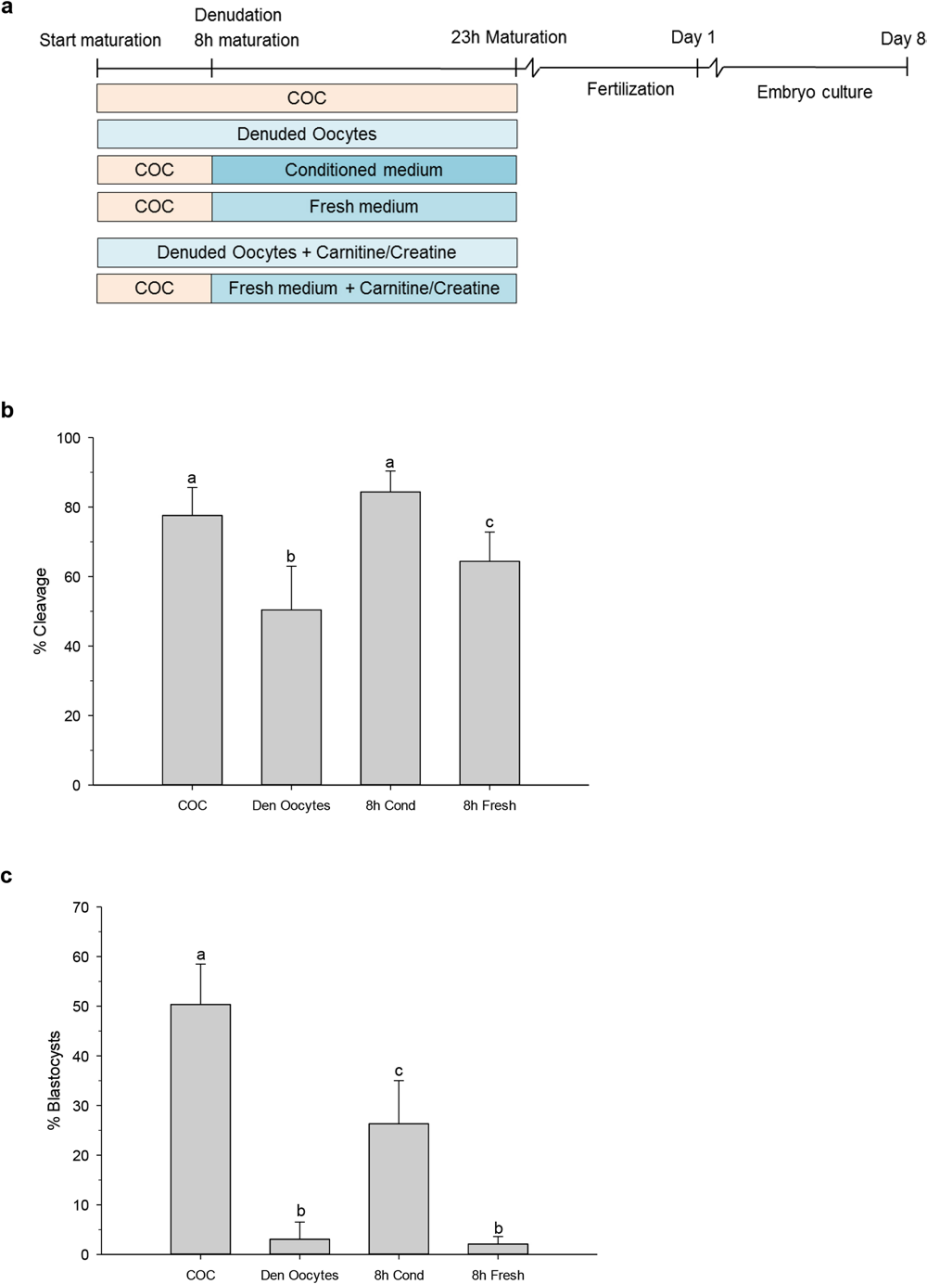
Experimental timeline (**a**) and developmental assessment after fertilization (**b**,**c**). (**a**) Intact COCs or denuded oocytes were matured for 23 h. Groups of intact COCs were denuded 8 h after the start of maturation and either placed back into their original medium or placed into fresh maturation medium. In addition, maturation media were supplemented with creatine or carnitine as indicated. After 23 h of maturation oocytes were fertilized and 5 days later cleavage was assessed; at day 8 after fertilization, blastocyst formation was assessed. Cleavage percentages of oocytes (**b**) and blastocyst formation of cleaved oocytes (**c**) matured differentially; as intact cumulus oocyte complexes (COC) for 23 h; as denuded oocytes (Den Oocytes) for 23 h; or matured as intact COCs for 8 h, denuded and placed back in their own conditioned medium (8 h Cond) or placed into fresh maturation medium (8 h Fresh). Data are presented as mean ± SD. Different letters above bars indicate outcomes that differ significantly (N = 6; P < 0.05).

To examine the influence of COC secretions on the acquisition of oocyte developmental competence during maturation, bovine oocytes were isolated from the ovaries of slaughtered cows and matured *in vitro* in four different groups: (1) 23 h as intact COCs; (2) denudation followed by 23 h IVM in the absence of cumulus cells; (3) 8 h IVM as COCs, followed by denudation and return to their own conditioned medium or (4) transfer to fresh medium for the remaining 15 h of IVM (Fig. 1a). After maturation, oocytes were fertilized *in vitro*, and the percentage that cleaved and developed to the blastocyst stage were determined on day 5 and 8 of culture, respectively.

The proportion of oocytes matured as COCs for 23 h that cleaved was 78%, whereas oocytes matured without cumulus were clearly compromised since cleavage was observed in only 50% (p < 0.001). Also, oocytes matured for 8 h, denuded and placed into fresh maturation medium showed a compromised cleavage of 64% (p = 0.001). However, when oocytes were matured within the COC for 8 h, denuded and placed back into conditioned medium, the proportion undergoing cleavage was similar (84%; p = 0.055) to that of oocytes matured as a COC for the entire 23 h (Fig. 1b).

The critical role of cumulus cells during oocyte maturation was confirmed by the difference in the proportion of oocytes matured within the COC that developed into blastocysts, as calculated from cleaved embryos, compared to oocytes matured without cumulus (50% and 3% respectively; p < 0.001). When oocytes were denuded after 8 h of maturation and placed back into COC-conditioned medium, the incidence of blastocyst formation was improved compared to oocytes transferred to fresh medium (26% and 2% respectively; p < 0.001; Fig. 1c). Whether conditioned medium from other cells, for example fibroblasts, oviductal cells or Buffalo Rat Liver cells can support preimplantation embryo development or would be beneficial for oocyte quality remains to be established. These data indicate that, in addition to direct contact between the oocyte and cumulus cells, factors secreted by COCs during the first 8 h of maturation are important for the acquisition of oocyte developmental competence during the remaining 15 h of maturation.

### Identification of factors secreted by COCs during oocyte maturation

To identify factors in cumulus cells and COC-conditioned medium that are important for acquisition or maintenance of oocyte developmental competence, cumulus cells were collected before the start of maturation (oocyte germinal vesicle stage: GV), and at 8 h and 23 h after the start of maturation and subjected to metabolic profiling using ultrahigh performance liquid chromatography-tandem mass spectroscopy. In addition, medium samples conditioned by COCs were collected after 8 h and 23 h. The metabolic profiles were compared to that of control (unconditioned) maturation medium.

To ensure that the cells remained metabolically active during maturation, cell viability was determined before and 23 h after *in vitro* maturation. The viability of the cumulus cells at 23 h of maturation was 112% (±19 SD) from that of cells at the beginning of maturation indicating that the cells remained viable throughout maturation. Additionally, the occurrence of apoptosis and necrosis in the COCs was analysed. During the first 8 h of maturation, almost no apoptosis or necrosis was detected. After 23 h of maturation, the levels of apoptosis had increased ~14%-fold representing about 10% of the cumulus cells, while almost no necrosis was detected.

**Figure 2:**
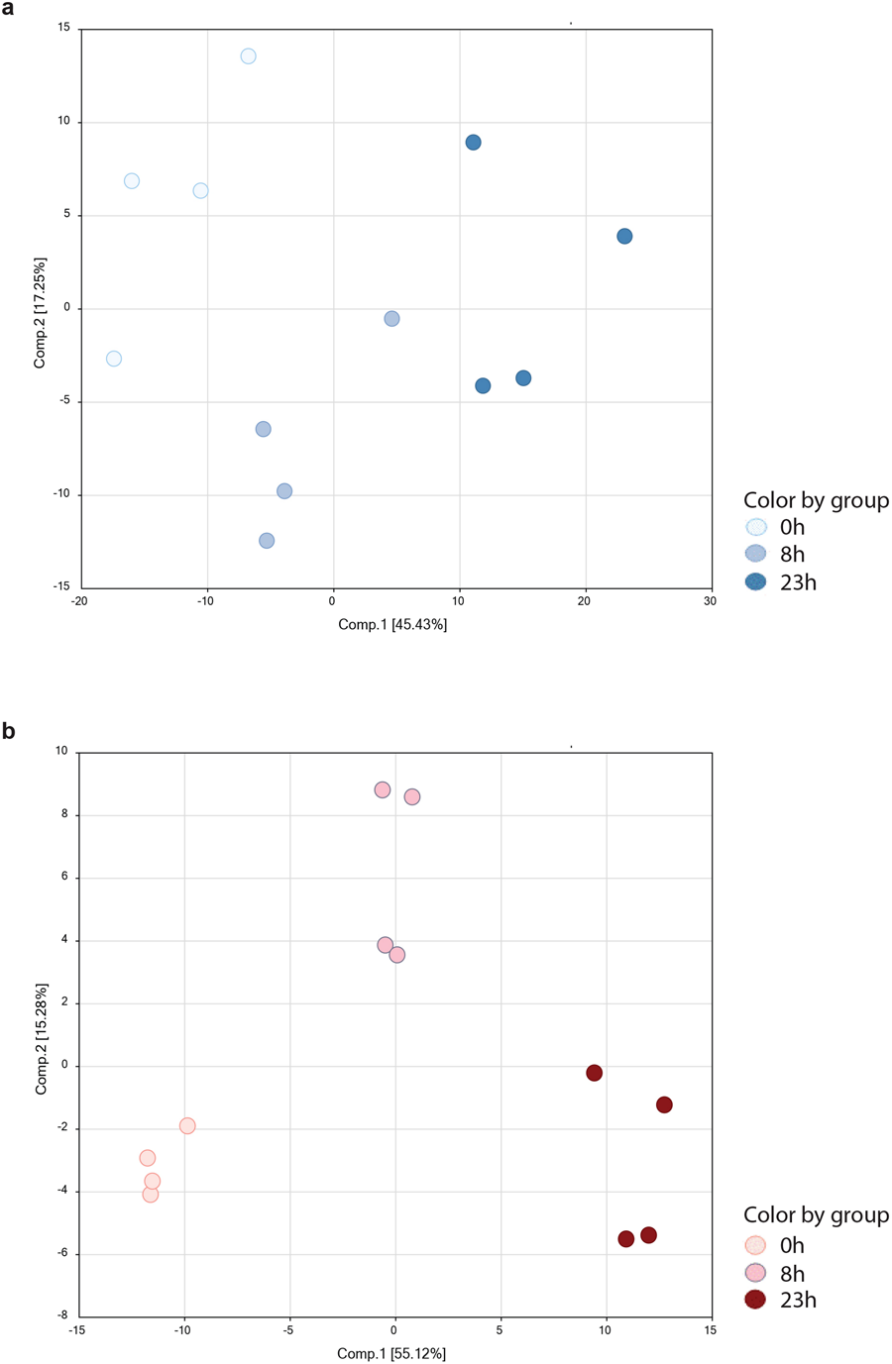
Principal component analyses for (**a**) bovine cumulus cells at GV stage (white), 8 h (light blue) and 23 h (blue) after start of maturation and (**b**) control maturation medium (0 h, light pink) and 8 h (dark pink) or 23 h (bordeaux) conditioning with maturing COCs.

Unsupervised principal component analyses (PCA) of the metabolic profiles of cumulus cells showed that the different groups cluster separately (Fig. 2a), indicating that the metabolites in cumulus cells changed over time in IVM. The differences between 8 and 23 h were less distinct however, possibly related to culture itself rather than duration. PCA of conditioned medium yielded three clearly separated clusters for the different time points (Fig. 2b) indicating that the nature of metabolites secreted by COCs into maturation medium also changed over time.

**Figure 3:**
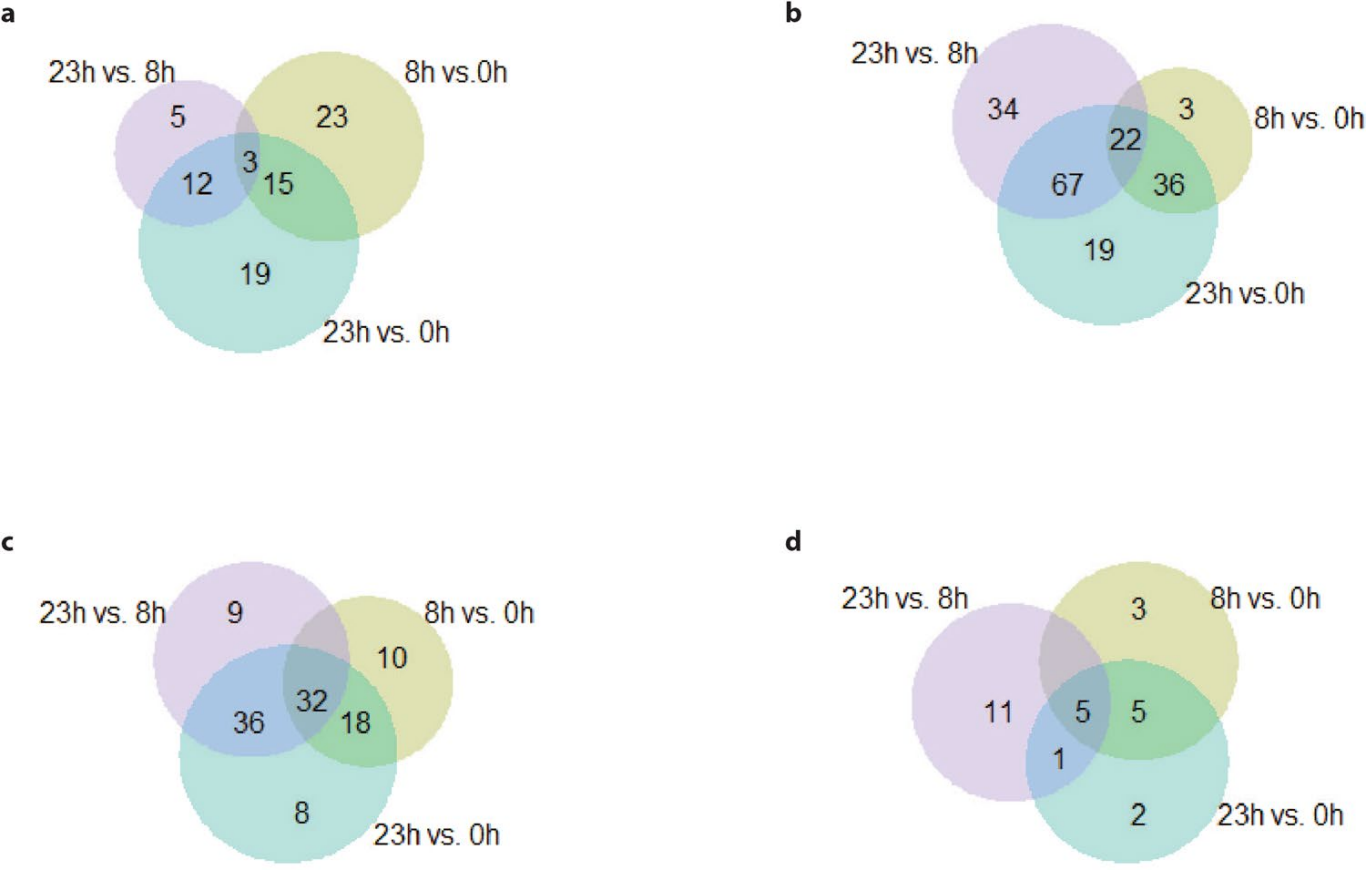
Venn diagram showing numbers of metabolites with significantly (Welch’s Two-Sample t-Test) altered concentrations in cumulus cells (**a**,**b**) and COC-conditioned medium (**c**,**d**) at different time points (8 h vs 0 h; 23 h vs 0 h and 23 h vs 8 h). (**a**) Increased and (**b**) decreased concentrations in cumulus cells. (**c**) Increased and (**d**) decreased concentrations in conditioned maturation medium.

In cumulus cells, 369 different biochemical components were detected. During the first 8 h of maturation, the levels of 41 of these components increased (Fig. 3a) and the levels of 61 decreased (Fig. 3b). Of these 41 and 61 components, 23 and three, respectively, changed exclusively during the first 8 h of maturation (Fig. 3a,b).

In COC-conditioned maturation medium, 173 different biochemical components were detected. Over the first 8 h of maturation, the levels of 60 biochemical components increased (Fig. 3c) whereas the levels of 13 decreased (Fig. 3d). The concentrations in conditioned medium of ten components increased exclusively during the first 8 h of maturation, whereas the levels of three factors decreased over the same time interval (Fig. 3c,d). Detected metabolites mentioned in the text below are presented in Tables 1 and 2 while an overview of all detected metabolites is presented in the supplementary data.

**Table 1:** List of biochemical components detected in cumulus cells.

Cumulus cells			Fold change	
Pathway	Sub Pathway	Biochemical Name	8 h vs. 0 h	23 h vs. 8 h
Amino Acid	Glycine, Serine, and Threonine Metabolism	betaine	*0.06*	1.40
		serine	1.18	*0.58*
	Glutamate Metabolism	glutamine	**3.03**	1.00
		pyroglutamine	*0.37*	**4.62**
	Lysine Metabolism	5-(galactosylhydroxy)-L-lysine	1.17	*0.55*
		2-aminoadipate	**2.21**	1.26
		N-trimethyl 5-aminovalerate	*0.08*	0.53
	Leucine, Isoleucine, and Valine Metabolism	leucine	**1.78**	1.73
		isoleucine	**2.48**	2.17
	Methionine, Cysteine, SAM, and Taurine Metabolism	N-acetylmethionine	0.98	*0.27*
		hypotaurine	**3.67**	1.52
		taurine	*0.04*	**4.80**
	Creatine Metabolism	guanidinoacetate	*0.46*	1.33
		creatine	*0.17*	*0.37*
		creatinine	*0.28*	*0.28*
	Glutathione Metabolism	glutathione, reduced (GSH)	1.49	*0.29*
		ophthalmate	*0.06*	0.71
Carbohydrate	Glycolysis, Gluconeogenesis, and Pyruvate Metabolism	3-phosphoglycerate	1.12	*0.02*
		phosphoenolpyruvate (PEP)	1.55	*0.02*
		pyruvate	1.23	**1.75**
		lactate	1.26	**1.91**
		glycerate	1.06	*0.25*
	Disaccharides and Oligosaccharides	lactose	*0.39*	*0.21*
	Nucleotide Sugar	UDP-glucose	**10.53**	0.35
		UDP-galactose	**8.74**	*0.20*
Energy	TCA Cycle	citrate	1.06	*0.11*
		aconitate [cis or trans]	1.18	*0.24*
		alpha-ketoglutarate	1.16	*0.52*
		fumarate	**1.81**	*0.37*
		malate	1.61	*0.20*
Lipid	Fatty Acid Metabolism(Acyl Carnitine)	acetylcarnitine (C2)	*0.07*	*0.31*
		octanoylcarnitine (C8)	*0.06*	0.53
		stearoylcarnitine (C18)	*0.38*	1.04
	Carnitine Metabolism	carnitine	*0.06*	1.25
	Phospholipid Metabolism	choline	1.05	*0.51*
		glycerophosphorylcholine (GPC)	**2.52**	*0.45*
		glycerophosphoethanolamine	**1.98**	*0.48*
	Phosphatidylcholine (PC)	1-palmitoyl-2-stearoyl-GPC (16:0/18:0)	*0.54*	*0.48*
		1-palmitoyl-2-alpha-linolenoyl-GPC (16:0/18:3n3)	*0.57*	*0.40*
		1,2-dilinoleoyl-GPC (18:2/18:2)	*0.50*	*0.43*
		1-linoleoyl-2-linolenoyl-GPC (18:2/18:3)	*0.41*	*0.49*
	Phosphatidylethanolamine (PE)	1,2-dipalmitoyl-GPE (16:0/16:0)	*0.59*	*0.62*
		1-palmitoyl-2-arachidonoyl-GPE (16:0/20:4)	*0.56*	*0.53*
		1-oleoyl-2-arachidonoyl-GPE (18:1/20:4)	*0.63*	*0.56*
	Phosphatidylserine (PS)	1-palmitoyl-2-oleoyl-GPS (16:0/18:1)	0.78	*0.62*
		1-stearoyl-2-oleoyl-GPS (18:0/18:1)	0.74	*0.44*
	Phosphatidylinositol (PI)	1-palmitoyl-2-arachidonoyl-GPI (16:0/20:4)	*0.58*	0.69
		1-stearoyl-2-oleoyl-GPI (18:0/18:1)	1.53	*0.49*
		1-stearoyl-2-arachidonoyl-GPI (18:0/20:4)	*0.56*	0.78
	Diacylglycerol	diacylglycerol (12:0/18:1, 14:0/16:1, 16:0/14:1) [2]	0.76	*0.59*
		palmitoyl-palmitoyl-glycerol (16:0/16:0) [2]	1.02	*0.43*
		linoleoyl-arachidonoyl-glycerol (18:2/20:4) [1]	1.00	*0.31*
		linoleoyl-arachidonoyl-glycerol (18:2/20:4) [2]	0.70	*0.18*
Lipid	Sphingolipid Metabolism	N-palmitoyl-sphinganine (d18:0/16:0)	**2.96**	1.26
		N-palmitoyl-sphingadienine (d18:2/16:0)	*0.34*	1.15
		tricosanoyl sphingomyelin (d18:1/23:0)	*0.68*	*0.53*
		sphingomyelin (d18:0/18:0, d19:0/17:0)	**9.99**	*0.55*
		hexadecasphingosine (d16:1)	*0.20*	1.03
		N-palmitoyl-heptadecasphingosine (d17:1/16:0)	*0.37*	1.01
	Ceramides	N-palmitoyl-sphingosine (d18:1/16:0)	*0.46*	1.19
		N-stearoyl-sphingosine (d18:1/18:0)	*0.40*	1.07
		ceramide (d18:1/17:0, d17:1/18:0)	*0.34*	1.08

Fold changes in component concentration were calculated for cumulus cells after 8 h maturation versus GV stage cumulus cells (0 h), and cumulus cells matured for 23 h versus 8 h. Cells marked in bold indicate a significant increase, and those marked in italic a significant decrease.

**Table 2:** List of biochemical components detected in cumulus-oocyte-complex-conditioned medium.

Maturation medium			Fold change	
Pathway	Sub Pathway	Biochemical Name	8 h vs. 0 h	23 h vs. 8 h
Amino Acid	Glycine, Serine, and Threonine Metabolism	glycine	1.05	**1.13**
		N-acetylglycine	1.06	**2.30**
		betaine	**6.80**	0.81
		serine	*0.79*	*0.61*
		N-acetylserine	**1.68**	**2.78**
		N-acetylthreonine	1.00	**2.75**
	Glutamate Metabolism	glutamate	0.99	**1.13**
		N-acetylglutamate	**2.92**	**2.43**
		N-acetylglutamine	**4.14**	1.46
		pyroglutamine	*0.42*	**1.35**
		S-1-pyrroline-5-carboxylate	*0.68*	1.17
	Leucine, Isoleucine, and Valine Metabolism	N-acetylleucine	1.11	***1.91***
		4-methyl-2-oxopentanoate	**7.55**	**2.28**
		3-methyl-2-oxovalerate	**4.92**	**2.66**
		3-methyl-2-oxobutyrate	1.69	**3.33**
		3-hydroxyisobutyrate	1.08	**5.58**
	Methionine, Cysteine, SAM, and Taurine Metabolism	N-acetylmethionine	2.77	**5.28**
		methionine sulfoxide	*0.45*	1.06
		hypotaurine	**35.65**	**2.54**
		taurine	**1.75**	0.84
	Urea cycle; Arginine and Proline Metabolism	urea	1.02	**9.51**
		ornithine	**13.39**	**2.88**
		2-oxoarginine	**1.81**	1.38
		dimethylarginine (SDMA + ADMA)	**6.92**	**3.63**
		trans-4-hydroxyproline	0.98	**1.05**
	Creatine Metabolism	guanidinoacetate	1.18	**3.16**
		creatine	**427.32**	1.21
		creatinine	**21.62**	0.95
	Glutathione Metabolism	cysteine-glutathione disulfide	1.32	**2.50**
		2-hydroxybutyrate/2-hydroxyisobutyrate	1.84	**2.15**
Carbohydrate	Glycolysis, Gluconeogenesis, and Pyruvate Metabolism	glucose	*0.59*	*0.17*
		pyruvate	1.15	**3.19**
		lactate	**667.34**	**1.84**
		glycerate	**1.43**	1.38
	Pentose Metabolism	ribose	*0.78*	1.09
		ribitol	**4.08**	**1.70**
		ribonate	**2.40**	1.07
		arabitol/xylitol	1.65	**2.02**
	Fructose, Mannose, and Galactose Metabolism	fructose	*0.52*	1.04
		mannitol/sorbitol	**5.90**	1.39
		mannose	1.03	*0.75*
	Aminosugar Metabolism	erythronate	1.22	**1.95**
		N-acetylglucosamine/N-acetylgalactosamine	1.00	**4.52**
Energy	TCA Cycle	citrate	**3.01**	**1.80**
		aconitate [cis or trans]	1.00	**1.96**
		alpha-ketoglutarate	**11.53**	**1.88**
		succinate	**3.66**	1.49
		fumarate	**1.58**	**2.90**
		malate	1.70	**4.17**
Lipid	Medium Chain Fatty Acid	heptanoate (7:0)	0.64	*0.77*
	Fatty Acid, Dicarboxylate	glutarate (pentanedioate)	0.68	**1.81**
		2-hydroxyglutarate	**1.78**	**1.82**
	Carnitine Metabolism	carnitine	**1.77**	0.89
	Inositol Metabolism	myo-inositol	**11.70**	*0.58*
	Phospholipid Metabolism	choline	*0.36*	0.88
		choline phosphate	**75.06**	1.40
		glycerophosphorylcholine (GPC)	1.21	**1.56**
		glycerophosphoethanolamine	1.21	**1.62**
		glycerophosphoinositol	1.27	**2.49**
	Glycerolipid Metabolism	glycerol	**6.89**	**1.47**
		glycerophosphoglycerol	**4.13**	**2.45**
	Mevalonate Metabolism	3-hydroxy-3-methylglutarate	**4.22**	**4.22**

Fold changes in biochemical component concentration were calculated for medium conditioned for 8 h versus unconditioned medium (0 h) and for medium conditioned for 23 h versus 8 h. Cells highlighted in bold exhibited a significant increase, and those highlighted in italic a significant decrease.

### Amino Acid Metabolism

Supplementation of amino acids to defined maturation medium has previously been shown to support the acquisition of development competence by the oocyte^13^. During maturation, the concentrations of nearly all detectable amino acids increased in the cumulus cells (Table S1 and Fig. S1); however, only a small number, including glutamine, leucine, and isoleucine increased significantly during the first 8 h of maturation (Table 1). Interestingly, while the concentrations of glutamine and leucine increased during the first 8 h of maturation, no further increase was observed during the remaining 15 h of maturation. The levels of all detectable amino acids had increased by 23 h of maturation (Table S1), with the exception of serine, the levels of which decreased between 8 h and 23 h of maturation. During the first 8 h of maturation, the levels of betaine, N-trimethyl 5-aminovalerate, taurine, and ophthalmate in cumulus cells decreased dramatically (Table 1) and their concentrations remained reduced throughout the remainder of the 23 h maturation (Table S1).

Changes in maturation medium conditioned by COCs for 8 h or 23 h were dominated by increases in the concentrations of various amino acids, including hypotaurine and ornithine. The concentrations of creatine, a substrate for amino acid production, increased 427-fold in maturation medium after 8 h (compared to 0 h) and remained increased (516-fold) until the end of IVM (23 h versus 0 h; Table S2). Creatine is derived from the reaction of guanidinoacetate, concentrations of which were not changed after 8 h of maturation, with S-adenosyl methionine. Creatine either reacts with ATP to yield the energy buffer, creatine phosphate (not detected), or is metabolised to the breakdown product creatinine, which was increased 22-fold in maturation medium after 8 h. In contrast to the levels of other biochemical components, a ten-fold enrichment of urea was detected, with much of the increase taking place in the second part of maturation. Overall, these data indicate an increase in amino acid synthesis and/or protein breakdown during IVM.

### Carbohydrate Metabolism

Glucose, lactose, and pyruvate are considered important supplements to oocyte maturation medium^14^. Glucose in the medium is consumed by the cumulus cells and converted into pyruvate, the preferred energy substrate of the oocyte.

During the first 8 h of maturation, concentrations of UDP-glucose and UDP-galactose in the cumulus cells increased more than other components of carbohydrate metabolism (Table S1 and Fig. S2); indeed, levels of detectable components of the carbohydrate categories glycolysis, gluconeogenesis, and pyruvate metabolism did not change during this period (Table 1). During anaerobic glycolysis, pyruvate is converted into lactate, the concentration of which increased significantly in cumulus cells during the final 15 h of maturation. During the entire 23 h IVM, the levels of most biochemical components of carbohydrate metabolism pathways, such as phosphoenolpyruvate (PEP) and 3-phosphoglycerate, decreased in cumulus cells (Table 1).

The concentration of glucose in maturation medium decreased significantly over time of IVM, indicating that it was consumed by the COCs. Surprisingly, during this period no significant change in the concentrations of lactate was detected in cumulus cells, whereas in the maturation medium the biggest increase among carbohydrate pathway components observed was for lactate; after 8 h, lactate concentrations had increased 667-fold (8 h versus 0 h) and after 23 h by 1227-fold (23 h versus 0 h; Table S2). In addition, increased levels of mannitol/sorbitol and ribitol were detected after 8 h. Pyruvate is the end product of glycolysis and was not present in unconditioned medium, but it was produced by the COCs and secreted into the maturation medium. An increased pyruvate concentration in the conditioned maturation medium was therefore detected during the second part of IVM (8–23 h) (Table 1 and Fig. S2).

Glucose is a central molecule in many metabolic pathways. Compared with cumulus cells collected at 0 h, the concentration of glucose did not change in cumulus cells after 8 h or 23 h of maturation, suggesting that glucose was immediately processed by cumulus cells to lactate or pyruvate, the preferred energy substrates for the oocyte.

### Energy Metabolism

Pyruvate is the end product of glycolysis and can enter the tricarboxylic acid cycle, an important route for ATP production. Cumulus cells matured for 8 h showed an increase in the concentration of fumarate, an intermediate of the tricarboxylic acid cycle, whereas the concentrations of other intermediates were not changed. By contrast, in cumulus cells matured for 23 h, the concentrations of citrate, aconitate, α-ketoglutarate, succinate, fumarate, and malate decreased (Table S1).

In conditioned medium, the concentrations of citrate, α-ketoglutarate, succinate, and fumarate were significantly increased after 8 h and 23 h. Indeed, the concentrations of nearly all detectable components of the tricarboxylic acid cycle increased in maturation medium during IVM. In particular, α-ketoglutarate and succinate accumulated in the maturation medium during the first 8 h, whereas during the second part of maturation the concentrations of fumarate and malate increased (Table 1).

The decrease in tricarboxylic acid cycle metabolites in the cumulus cells indicates reduced pyruvate oxidation in the mitochondria of those cells. Additionally, intermediates of the tricarboxylic acid cycle were secreted into the maturation medium, where they might be available for other reactions in follicular cells; for example, malate can be used for amino acid synthesis.

### Lipid metabolism

The main lipid metabolic pathways can be subdivided into different specific systems, such as the phosphatidylcholine and phosphatidylinositol pathways. In general, decreased levels of biochemical components of phospholipid, phosphatidylcholine, phosphatidylethanolamine, phosphatidylserine, phosphatidylinositol, diacylglycerol, sphingolipid, and ceramide metabolism were detected in cumulus cells (Table S1 and Fig. S3). During maturation, concentrations of most of the lipids decreased in cumulus cells by 8 h and 23 h, among which components of the phosphatidylcholine and ceramide pathways showed a more prominent decline during the first 8 h. The biochemical components with the lowest fold change in cumulus cells were acetylcarnitine, octanylcarnitine, hexanoylcarnitine, and carnitine. Over the 23 h of maturation, the concentrations of various components of phosphatidylcholine, phosphatidylethanolamine, and sphingolipid metabolism decreased in cumulus cells (Table S1).

In COC-conditioned medium, increased levels of carnitine, myo-inositol, cholinephosphate, and glycerol were detected after 8 h of maturation. The comparison between 8 h and 23 h demonstrated a decrease in the concentrations of myo-inositol, indicating that myo-inositol was consumed during the last 15 h of maturation (Table 1 and Fig. S3). Carnitine is an important factor during β-oxidation, and its concentrations in cumulus cells decreased after 8 h of maturation (16.7-times); the levels of its derivatives also decreased sharply in cumulus cells during the first 8 h of maturation. Furthermore, during 8 h of conditioning the amount of carnitine in the maturation medium increased 1.8-fold.

These data show that, in cumulus cells, phospholipids are broken down to yield phospholipid precursors which are excreted, possibly for use by the oocyte.

### Carnitine and creatine supplementation

Carnitine and creatine were among the biochemical components that were elevated in COC-conditioned medium after 8 h. In order to better understand their functions, oocytes were matured in the presence of these components of lipid metabolism (carnitine) or the amino acid pathway (creatine). Oocytes were denuded and matured for 23 h or denuded 8 h after the onset of maturation and subsequently placed into fresh maturation medium supplemented with carnitine or creatine (Fig. 1a).

**Figure 4:**
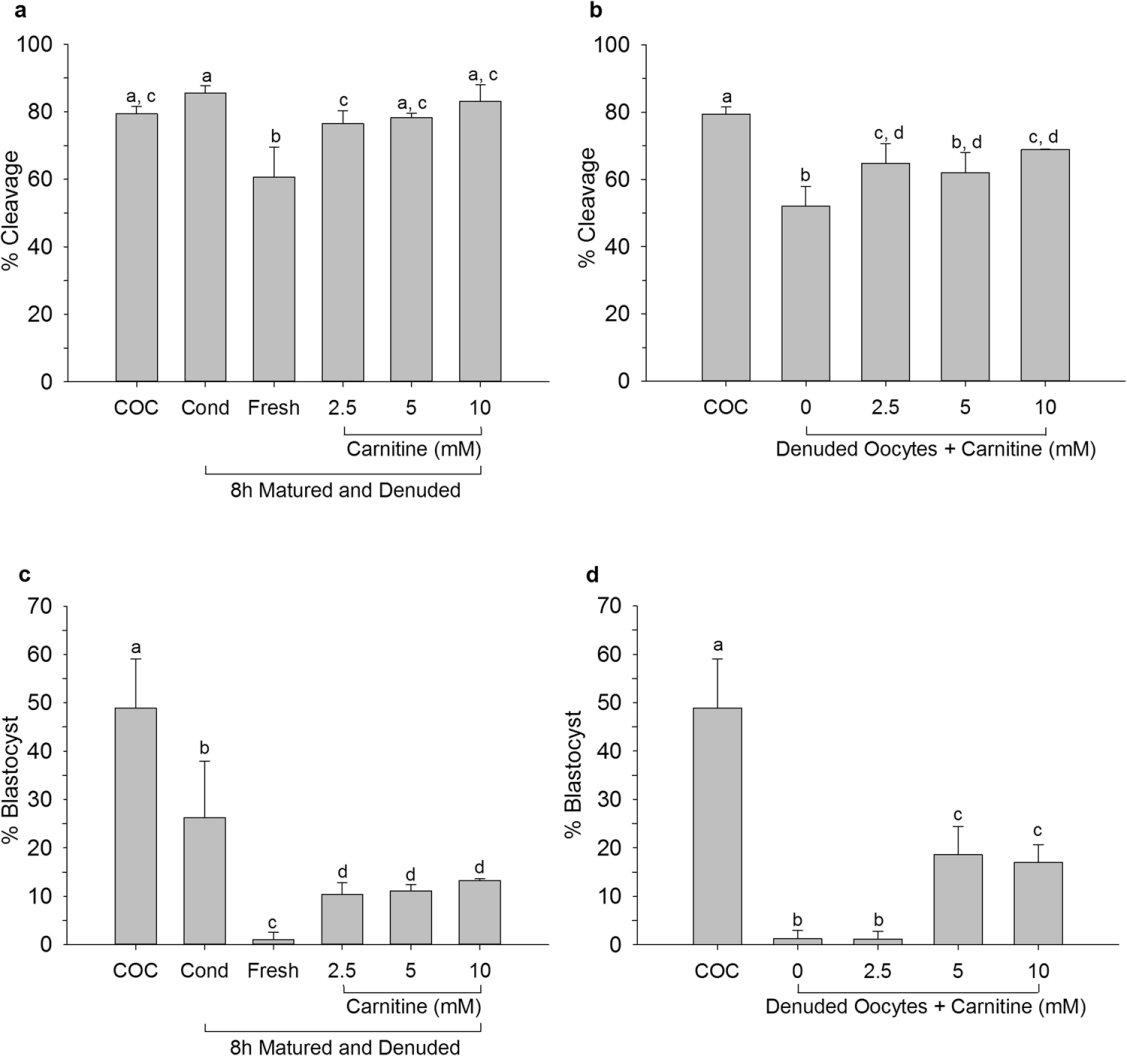
Cleavage of oocytes (**a**,**b**) and blastocyst formation of cleaved oocytes (**c**,**d**) matured in carnitine supplemented medium. Oocytes were matured as intact COCs for 23 h (COC) or as intact COCs for 8 h before being denuded and placed back in their own conditioned medium (Cond), or transferred to fresh maturation medium (Fresh) or to fresh maturation medium supplemented with varying concentrations of carnitine. Denuded oocytes were matured for 23 h in conventional medium or for 23 h in carnitine-supplemented medium. Data are presented as mean ± SD. Data points labelled with the same letter did not differ significantly (N = 3; P < 0.05).

The proportion of oocytes that had cleaved 5 days after fertilization was decreased for oocytes denuded after 8 h of maturation and placed into fresh maturation medium compared to control COCs; however, supplementation of the maturation medium with carnitine resulted in cleavage rates similar to the control (Fig. 4a). Most importantly, denuded oocytes matured in maturation medium supplemented with 2.5 or 10 mM carnitine yielded better cleavage rates than denuded oocytes matured without carnitine supplementation (Fig. 4b). Percentages of oocytes that developed to blastocysts after fertilization were also increased after carnitine supplementation, compared to denuded oocytes placed into fresh maturation medium after 8 h of maturation (Fig. 4c) and oocytes matured for 23 h in the absence of cumulus cells (Fig. 4d). Combined, these data demonstrate the importance of carnitine for the acquisition of developmental competence by a denuded oocyte during *in vitro* maturation.

**Figure 5:**
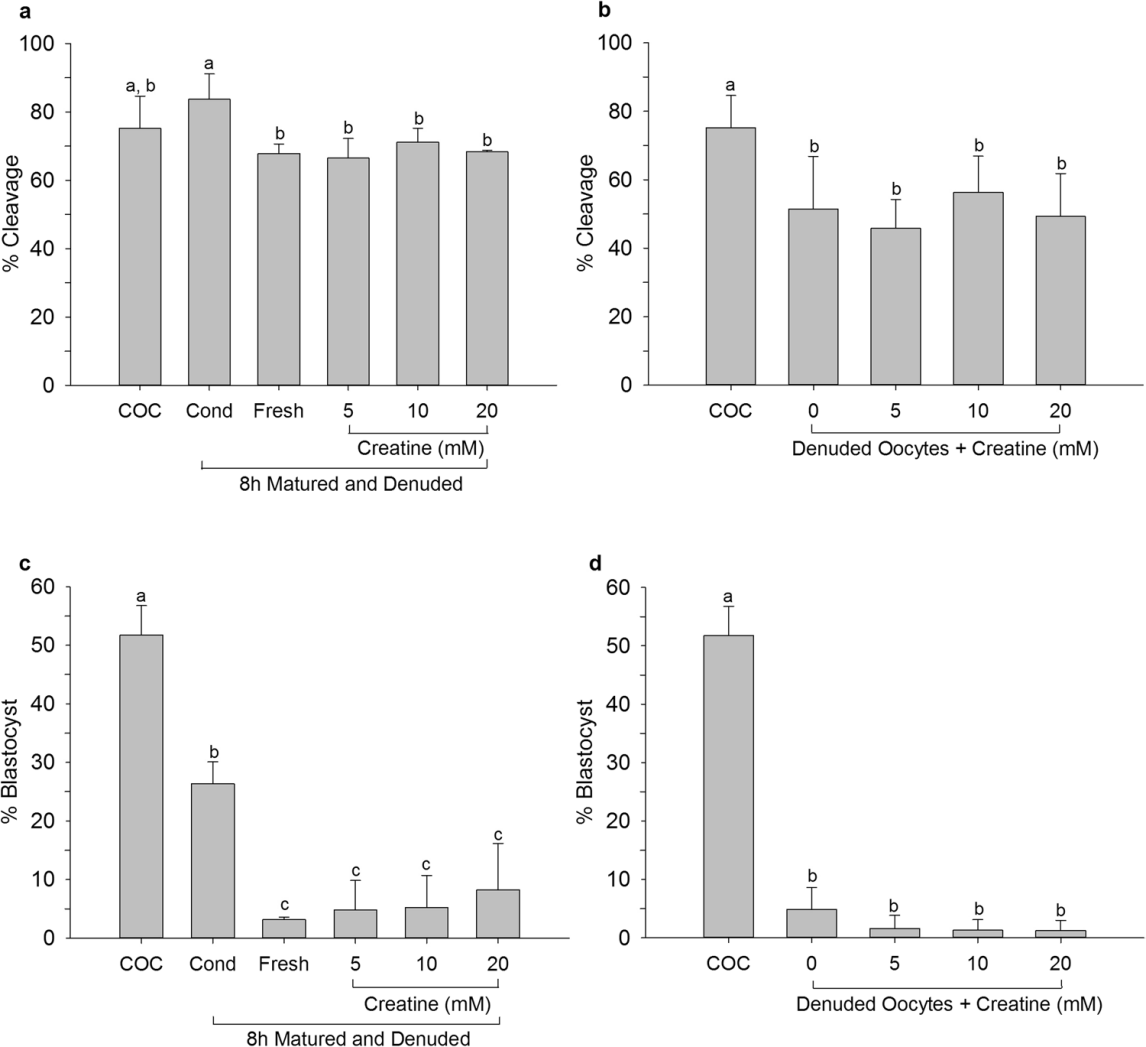
Cleavage of oocytes (**a**,**b**) and blastocyst formation of cleaved oocytes (**c**,**d**) matured in creatine supplemented medium. Oocytes were matured as intact COCs for 23 h (COC) or as intact COCs for 8 h before being denuded and placed back in their own conditioned medium (Cond), transferred to fresh maturation medium (Fresh) or into fresh maturation medium supplemented with varying concentrations of creatine. Denuded oocytes were matured for 23 h or for 23 h in creatine-supplemented medium. Data are presented as mean ± SD. Data points labelled with the same letter did not differ significantly (N = 3; P < 0.05).

Success of cleavage for oocytes matured for 8 h, denuded and placed into creatine supplemented maturation medium were the same as those for similar oocytes placed in fresh medium (Fig. 5a). Similarly, creatine supplementation of maturation medium did not significantly alter the percentage of oocytes that cleaved after maturation for 23 h without cumulus cells (Fig. 5b). Blastocyst formation for oocytes denuded after 8 h or matured for the entire 23 h without cumulus cells was also not improved by creatine supplementation (Fig. 5c,d). These data suggest that creatine alone does not affect the developmental competence of the oocyte during maturation.

## Discussion

Oocyte developmental competence is influenced by direct contact with cumulus cells, and by the microenvironment created by the cumulus-oocyte-complex during maturation. The importance of cumulus cells for oocyte maturation and the acquisition of developmental competence has been reported previously^1,5^. Here, we examined the metabolic profile of cumulus cells at different time points during maturation, and that of COC-conditioned medium. An increase in the occurrence of apoptosis occurred between 8 and 23 h of maturation as has been reported previously^15^. The numbers of viable cells remained similar however during the whole maturation period, suggesting that proliferation compensated for apoptosis.

Our results show that, during the first 8 h of maturation, a microenvironment is created that supports oocyte developmental competence. If oocytes were matured for 8 h within intact COCs, denuded and placed back into their conditioned medium, 26% developed to blastocysts, compared to only 2% if the oocytes were placed back into fresh (non-conditioned) maturation medium. This demonstrates that COCs secrete factors during the first 8 h of maturation that are beneficial for oocyte acquisition of developmental competence during the following 15 h of maturation.

To identify candidate metabolites that improve oocyte maturation, a UPLC-MS/MS analysis was performed. It transpired that the concentrations of 77 different biochemical components increased and 181 decreased in the cumulus cells (Fig. 3a,b), whereas the concentrations of 113 metabolites increased and 27 decreased (Fig. 3b,d) in the maturation medium during all analysed time points. In total more compound species were detected in cumulus cells than in conditioned medium. Since detection levels were similar between media and cells this indicates that indeed more components were present in cumulus cell than secreted into the medium. Most probably, the majority of changes in metabolite concentrations result from the general metabolic activity of the cumulus cells, nevertheless the IVM experiments demonstrate that cumulus cells also secrete factors that assist the oocyte to successfully complete the maturation process. In theory, decreased component levels in condition medium could also have resulted from degradation of metabolites in the medium, independent of the presence of the oocyte and cumulus cells between 8 and 23 h of maturation.

### Amino acid metabolism

The increase in concentration for most of the amino acids in both the cumulus cells and medium probably reflects normal metabolism. One exception was the decrease in cellular serine concentrations during the final 15 h of maturation, and a decrease in concentrations in the conditioned medium throughout. Cancer cells deprived of serine have been reported to undergo oxidative stress^16^. It is possible that oocytes similarly require serine from the environment to reduce oxidative stress by channelling depleted serine stores to glutathione synthesis, as cancer cells do^17^. Alternatively, serine may be used for one-carbon metabolism^16^ to generate one-carbon units for nucleotide synthesis, e.g. glycine or α-ketoglutarate.

In bovine oocytes, oxidative metabolism is the major mechanism responsible for energy production^18^. Bovine cumulus cells exhibit high alanine aminotransferase and aspartate aminotransferase activity, which is presumably linked to amino acid synthesis^19^. In addition it has been suggested that aspartate has an important role in oocyte energy metabolism, because it can be converted into oxaloacetate for use in the tricarboxylic acid cycle^19^. Indeed, our results show a significant increase in aspartate concentrations in cumulus cells at all time points tested (see also Table S1); however, concentrations did not change in the surrounding media during maturation, suggesting that aspartate is used directly for energy production in the cumulus cells or is transferred to the oocyte. Supplementation of maturation media with aspartate or alanine has been reported to improve the maturation of denuded oocytes, indicating their importance as oxidative substrates for the oocyte^19^ or in the synthesis of protein factors involved in meiotic maturation. However, in our study no IVM-related changes in alanine concentrations were detected in cumulus cells, and only a small increase between 8 h and 23 h of maturation was detected in conditioned medium.

### Carbohydrate metabolism

It has been demonstrated that cumulus cells can metabolize glucose into pyruvate, and deliver the latter to the oocyte^20,21^. Pyruvate can be directly transferred via gap junctions in the transzonal processes from the cumulus cell to the oocyte, but it can also be secreted by the cumulus cells and taken up from the surrounding medium by the oocyte^22^. Indeed, in conditioned medium the concentrations of both pyruvate and lactate increased, while glucose concentrations decreased by 23 h of maturation. In particular, the lactate concentration of maturation medium increased 667-fold after 8 h IVM, whereas in cumulus cells an increase was detected during the final 15 h of maturation. During the first 8 h of maturation, there was no significant change in pyruvate in the cumulus cells or maturation medium, suggesting that during the first 8 h pyruvate is channelled to additional metabolic pathways such as the tricarboxylic acid cycle. Indeed, a significant increase in tricarboxylic acid cycle metabolites, such as citrate and α-ketoglutarate was detected. Taken together these data indicate that glucose is utilized for pyruvate production, especially during the first 8 h of maturation, and that the pyruvate is taken up by the oocyte as an energy substrate. Alternatively, pyruvate could be converted into lactate. Indeed, oocytes are rich in members of the SLC16A (MCT) family of monocarboxylic acid transporters^23^ that can transport lactate. In addition, supplementation of maturation medium with lactate and NAD has been reported to improve maturation of denuded bovine oocytes^24^.

Interestingly, between 8 h and 23 h of IVM the concentrations of almost all detected components of the tricarboxylic acid cycle decreased in cumulus cells. By contrast, the medium showed a temporally related increase in these components. Other studies have reported that culture of denuded bovine oocytes with intermediates of the tricarboxylic acid cycle, oxaloacetate or malate, in combination with NAD, a coenzyme important for the redox status of the cell, resulted in a significant improvement in the proportion reaching MII^19^. By contrast, no such improvement was detected when oocytes were cultured as COCs^19^. Together these data suggest that biochemical processes in cumulus cells can influence the redox state of the oocyte. This is important for further development, because excess reactive oxygen species generation compromises the oocyte’s developmental competence.

Increased concentrations of nucleotide sugars, including UDP-glucose, were detected in cumulus cells, especially after the first 8 h of maturation. UDP-glucose is involved in synthesis of hyaluronan, an element of the cumulus cell matrix, suggesting that UDP-glucose is involved in cumulus cell matrix changes during maturation. Indeed, an increase in UDP-glucose and UDP-galactose was evident only during the first 8 h of maturation, the critical period for cumulus expansion.

### Lipid metabolism

Lipids were mostly detected in cumulus cells and, in general, their concentrations decreased during maturation while in the medium much fewer changes occurred over time. The concentrations of some components of phospholipid metabolism, like choline phosphate, glycerophosphoethanolamine, and glycerophosphoinositol increased in the medium, however, suggesting that they were generated by the COCs. Lipid droplets have also been found in cumulus cells and β-oxidation seems to take place predominantly in the cumulus cells^25,26^. Energy production, in terms of ATP generation, is much higher for β-oxidation than glycolysis^25^, but it was not known whether fatty acids from the follicular fluid were used to generate ATP. Supplementation of maturation medium with linolenic acid has been reported to have a positive effect on oocyte maturation and embryo development^27^ and was able to neutralise the detrimental effects of palmitic acid and stearic acid on *in vitro* maturation^28^. Furthermore, cumulus cells regulate the transport of fatty acids to the oocyte, after exposure to elevated free fatty acids cumulus cells stored these lipids^29^. Cholesterol biosynthesis also requires oocyte-cumulus cell cooperation^30^, since transcripts important for cholesterol synthesis pathways have been found in cumulus cells, but not in oocytes^31,32^. This indicates that, in terms of energy production, lipids are important during oocyte maturation and establishment of a developmentally competent oocyte. The concentrations of various lipid classes, such as phosphatidylcholine and phosphatidylethanolamine, decreased in cumulus cells during maturation, however they were not detected in the maturation medium, suggesting metabolic cooperation in lipid metabolism between the cumulus cells and the oocyte. Whether the changes observed in the *in vitro* matured cumulus cells also apply to those from *in vivo* matured COCs remains to be established.

### Carnitine and creatine

Of the components that decreased in cumulus cells and increased in medium, the influence of carnitine and creatine, but no other detected biochemicals, were tested further. Creatine was chosen for its impressive concentration increase in the medium within 8 h. Carnitine was chosen because its concentration also increased in the medium but more importantly because in cumulus cells the levels of carnitine metabolites (acetylcarnitine, hexanoylcarnitine and octanoylcarnitine) decreased, suggesting that they are further metabolised and this pathway is important for energy production.

While creatine supplementation had no effect, supplementation with carnitine improved the developmental competence of denuded oocytes. This effect was also detected, if oocytes having a low developmentally potential^33^, which were retrieved from small antral follicles (2–5 mm), or juvenile oocytes^34^ were cultured with L-carnitine.

Pig oocytes matured in glucose free maturation medium supplemented with carnitine, showed accelerated nuclear maturation and an enhanced ability to reach the MII stage^35^. A study in camels also showed a positive effect of carnitine supplementation on oocyte nuclear maturation, as well as on the developmental potential of the oocytes^36^. All of which supports the hypothesis that fatty acid metabolism is important during oocyte maturation. In addition, carnitine has antioxidant activity which has been reported to protect the DNA against fragmentation due to reactive oxygen species generation^35,37^. An example for antioxidants is glutathione, whose levels were increased after carnitine supplementation^38,39^.

Creatine supplementation did not improve blastocyst production. This was surprising because creatine can enter the adenosine salvage pathway^40^ to provide ATP for the oocytes and the enzyme creatine kinase was associated with ATP supply during spindle formation in 2- and 4-cell mouse embryos^41^. Possibly, energy storage and transportation is more established during the later stages of maturation, since the phosphocreatine concentration in cumulus cells was decreased after 23 h of maturation.

In summary, numerous metabolic processes occur in the cumulus cells during the first 8 h of IVM that help to create a beneficial microenvironment for the maturing oocyte, which needs to store energy and other small molecules to ensure that it remains metabolically active until embryonic genome activation takes place.

## Materials and Methods

All chemicals were obtained from Sigma-Aldrich (St. Louis, Missouri, USA), unless otherwise stated.

### Collection of ovaries, harvesting COCs, *in vitro* maturation and fertilization

Bovine ovaries were obtained from a local slaughterhouse and transported in a thermos flask to the laboratory. On arrival at the laboratory, the ovaries were rinsed in tap water and held at 30 °C in physiological saline (0.9%) supplemented with 100 IU penicillin and 100 µg streptomycin per ml. COCs were aspirated from small antral follicles (2–8 mm) using an 18-gauge needle connected to a low pressure vacuum pump via a 50 ml collection tube, as described by van Tol *et al*.^42^. Only oocytes with multiple layers of cumulus cells were selected for *in vitro* maturation, and rinsed with HEPES buffered M199 (Gibco BRL, Paisley, UK). The obtained COCs were randomly distributed into groups of 50–60 in 4-well culture plates (Nunc A/S, Roskilde, Denmark) containing 500 µl NaHCO_3_-buffered M199 (Gibco BRL) supplemented with 1% (v/v) penicillin-streptomycin (Gibco BRL), 0.02 IU FSH/ml (Sioux Biochemical Inc., Sioux Centre, IA, USA), 0.02 IU LH/ml (Sioux Biochemical Inc.), 7.7 µg/ml cysteamine, and 10 ng/ml epidermal growth factor. Maturation involved culture for 23 h at 38.5 °C in a humidified atmosphere containing 5% CO_2_.

For *in vitro* fertilization, frozen thawed sperm were selected by centrifugation through a discontinuous percoll (90/45%) gradient. Spermatozoa were added at a final concentration of 1 × 10^6^ cells/ml to fertilization medium^43^ supplemented with 1.8 IU/ml heparin, 20 µM d-penicillamine, 10 µM hypotaurine, and 1 µM epinephrine. Fertilization incubation was performed for 18–22 h at 38.5 °C in a humidified atmosphere containing 5% CO_2_.

Presumptive zygotes were vortexed for 1 min to remove adhered sperm, transferred to synthetic oviductal fluid (SOF)^42^ and cultured at 39 °C in a humidified atmosphere, containing 5% CO_2_ and 7% O_2_. On day 5 of culture, cleaved embryos only were transferred to a new well containing fresh SOF, and cultured further until day 8.

### Creatine and carnitine supplementation

To examine the effects of carnitine and creatine during oocyte maturation, various concentrations of either were added to the maturation medium. The final concentrations used were 2.5 mM, 5 mM, and 10 mM L-Carnitine-hydrochloride and 5 mM, 10 mM, and 20 mM Creatine-monophosphate. Two different groups of oocytes were exposed to these concentrations of carnitine or creatine; first, oocytes matured as intact COCs for 8 h, that were vortexed for 3 min to remove the cumulus cell, before being returned to fresh maturation medium supplemented with creatine or carnitine or, second, oocytes denuded before the start of maturation and subsequently matured for 23 h with creatine or carnitine in the medium.

Control groups included intact COCs and denuded oocytes matured for 23 h, and oocytes matured with an intact cumulus for 8 h, and then denuded by vortexing for 3 min before being returned to their original conditioned medium or transferred to fresh maturation medium. Each experiment was performed three times independently.

### Collection of cumulus cell and maturation media

Cumulus cells were collected immediately after COC isolation for germinal vesicle stage oocytes, 8 h and 23 h after the start of *in vitro* maturation. COCs were denuded by recurrent pipetting through a narrow pipette, followed by vortexing and centrifugation.

Conditioned media were collected after 8 h and 23 h of COC culture. Media and cumulus cells were snap frozen in liquid nitrogen and stored at −80 °C until further analysis. Cells and media were from four biologically independent experiments.

### Viability assay

To assess the viability of cumulus cells, ten wells each with 15 COCs were cultured in 100 µl maturation medium in a 96-well plate (Nunc A/S) and incubated at 38.5 °C. To determine the activity, 10 µl AlamarBlue solution (Thermo Fisher, Waltham, MA, USA) was added either directly or after 21 h of maturation and cells were cultured for an additional 2 h. Fluorescence was subsequently measured with a DTX 880 Multimode Detector (Beckman Coulter, Woerden, The Netherlands) with an excitation wavelength of 535 nm and an emission wavelength of 590 nm. The sample signals were background corrected and viability determined after 23 h as percentage of the fluorescence values determined at the beginning of maturation. This experiment was performed three times independently on different days, with 10 replicates of 15 COCs each.

### Apoptosis

A Cell Death detection ELISAPlus kit (Roche, Basel, Switzerland) was used to detect apoptosis and necrosis. Groups of 15 COCs were cultured in 100 µl maturation medium in a 96-well plate in two independent experiments and cells and media prepared according to the manufacturer’s instructions. Absorbance was measured with a DTX 880 Multimode Detector (Beckman Coulter).

### Metabolic analysis

Sample preparation, ultrahigh performance liquid chromatography-tandem mass spectroscopy (UPLC-MS/MS) and bioinformatics analysis were performed by Metabolon Inc. (Durham, NC, USA)^44^. For missing values, minimum value imputation was used to calculate the fold-changes.

Briefly, the MicroLab STAR^®^ system from Hamilton Company was used to prepare samples for four different analytical methods. The samples were analysed by two separate reverse phase (RP/UPLC)-MS/MS methods using positive ion mode electrospray ionization, one involving RP/UPLC-MS/MS with negative ion mode electrospray ionization, and one involving hydrophilic interaction chromatography (HILIC)/UPLC-MS/MS with negative ion mode electrospray ionization.

Raw data was extracted, peak-identified, and QC processed using Metabolon’s hardware and software.

### Statistical analysis

Statistical analysis of cleavage and blastocyst formation was performed using SPSS version 24 (SPSS Inc., Chicago, Il, USA). A logistic regression for grouped data was used to analyse success of cleavage and blastocyst formation, with condition and experimental run included as fixed factors. Data are presented as mean ± SD. A *P* value < 0.05 was considered to be statistically significant.

Welch’s two-sample t-test was used two compare metabolite concentrations in the cumulus cells and medium at different time points.

### Data availability

The datasets generated and analysed during the current study are available from the corresponding author on reasonable request.

## Electronic supplementary material

Supplementary information
