# Supplementary material for: Metabolomic profiles of bovine cumulus cells and cumulus-oocyte-complex-conditioned medium during maturation in vitro
Source: Sci Rep. 2018 Jun 21;8:9477. doi: 10.1038/s41598-018-27829-9 (PMC6013446; doi:10.1038/s41598-018-27829-9)
Supplement: Supplementary file 1 — Supplementary information [file 41598_2018_27829_MOESM1_ESM.pdf]

Supplementary Information

Metabolomic profiles of bovine cumulus cells and cumulus-oocyte-complex-conditioned medium during maturation *in vitro*

Karen Uhde[1], Helena T.A. van Tol[1], Tom A.E. Stout[1,2] and Bernard A.J. Roelen*[1,2]

[1] Department of Farm Animal Health, Faculty of Veterinary Medicine, Utrecht University, Utrecht, The Netherlands

[2] Department of Equine Sciences, Faculty of Veterinary Medicine, Utrecht University, The Netherlands

Figure S1: Cytoscape specific pathway visualisation of selected components of the amino acid pathway

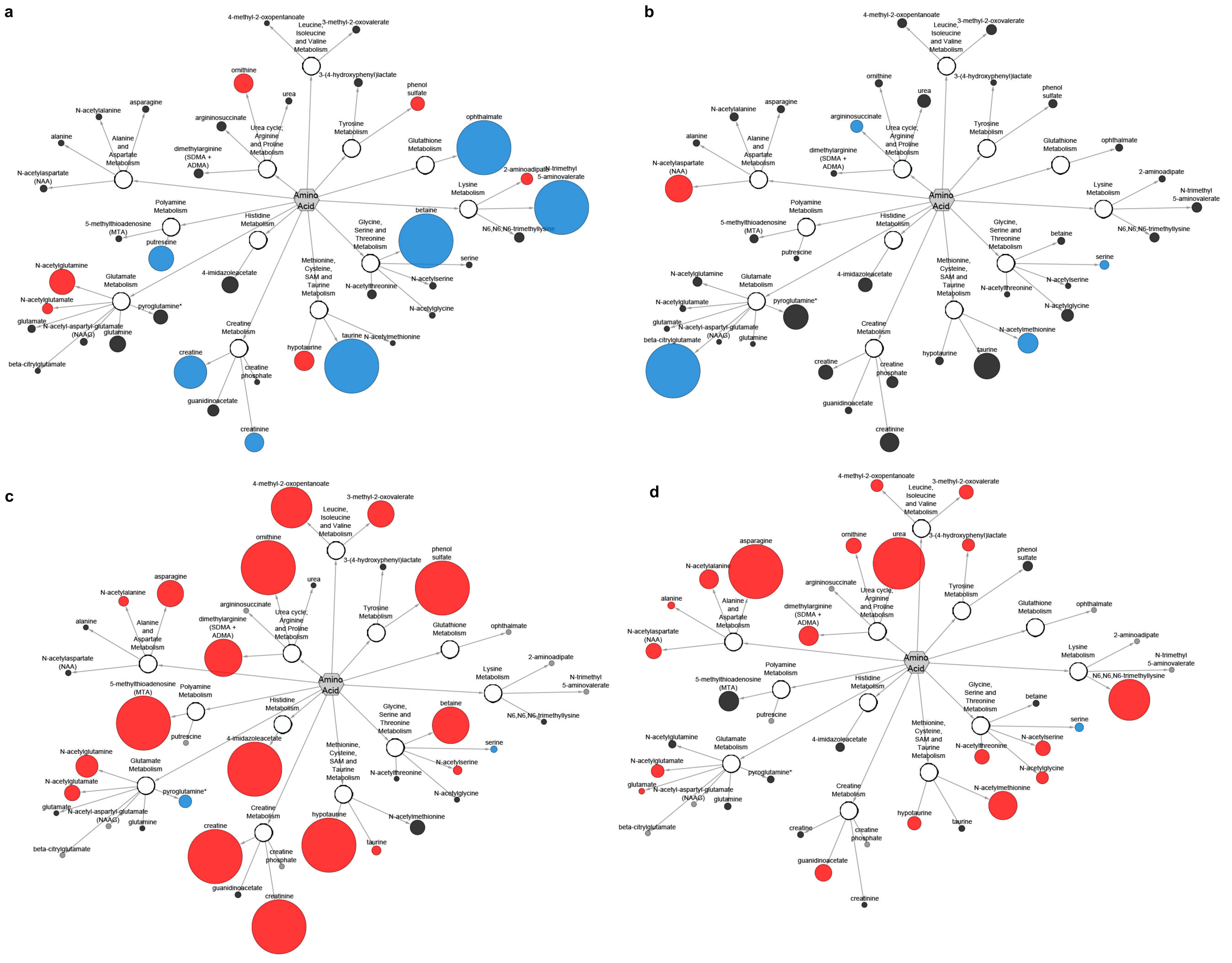

Figure S2: Cytoscape specific pathway visualisation of selected componentsof the carbohydrate pathway.

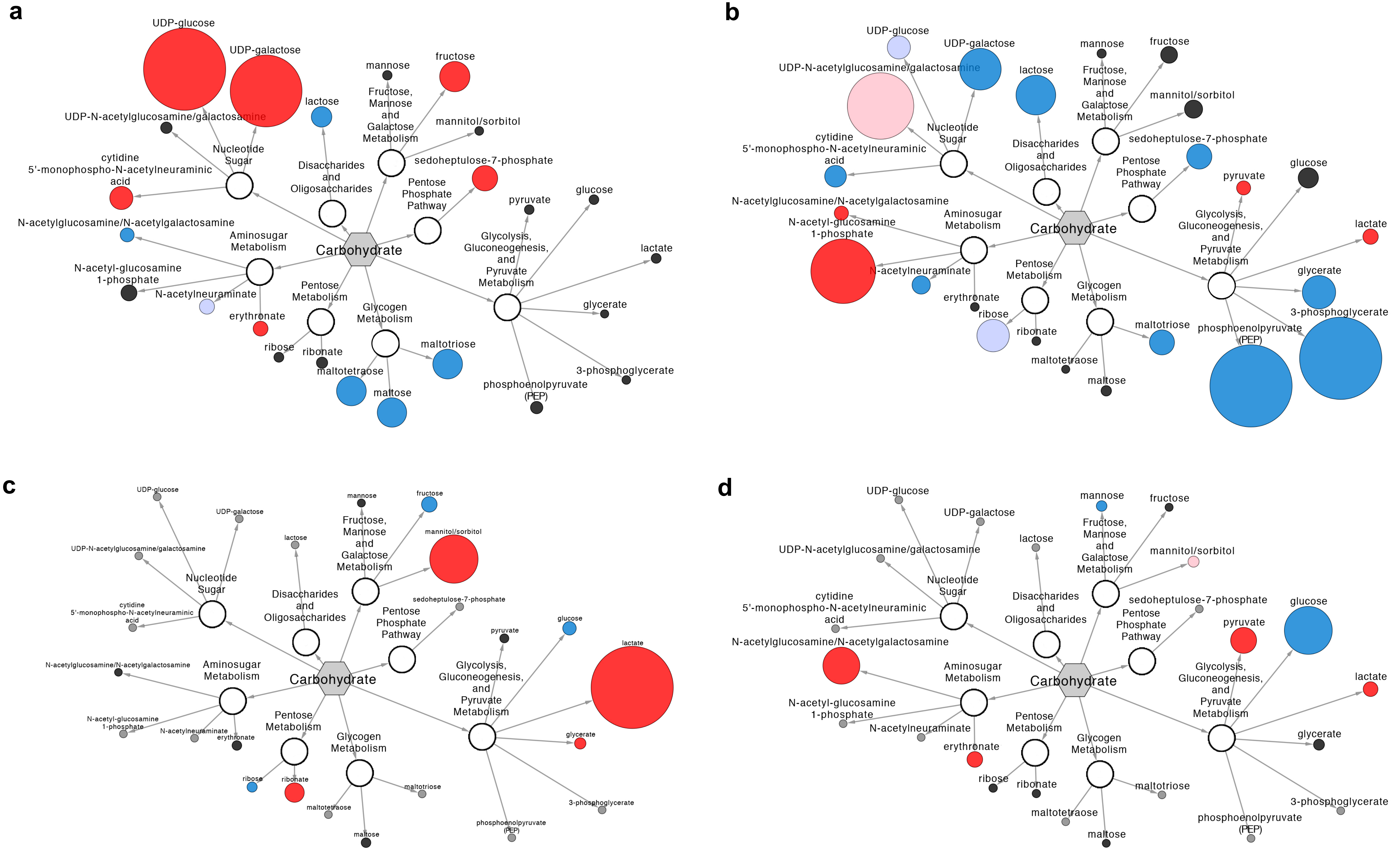

Figure S3: Cytoscape specific visualisation of selected components of the lipid pathway

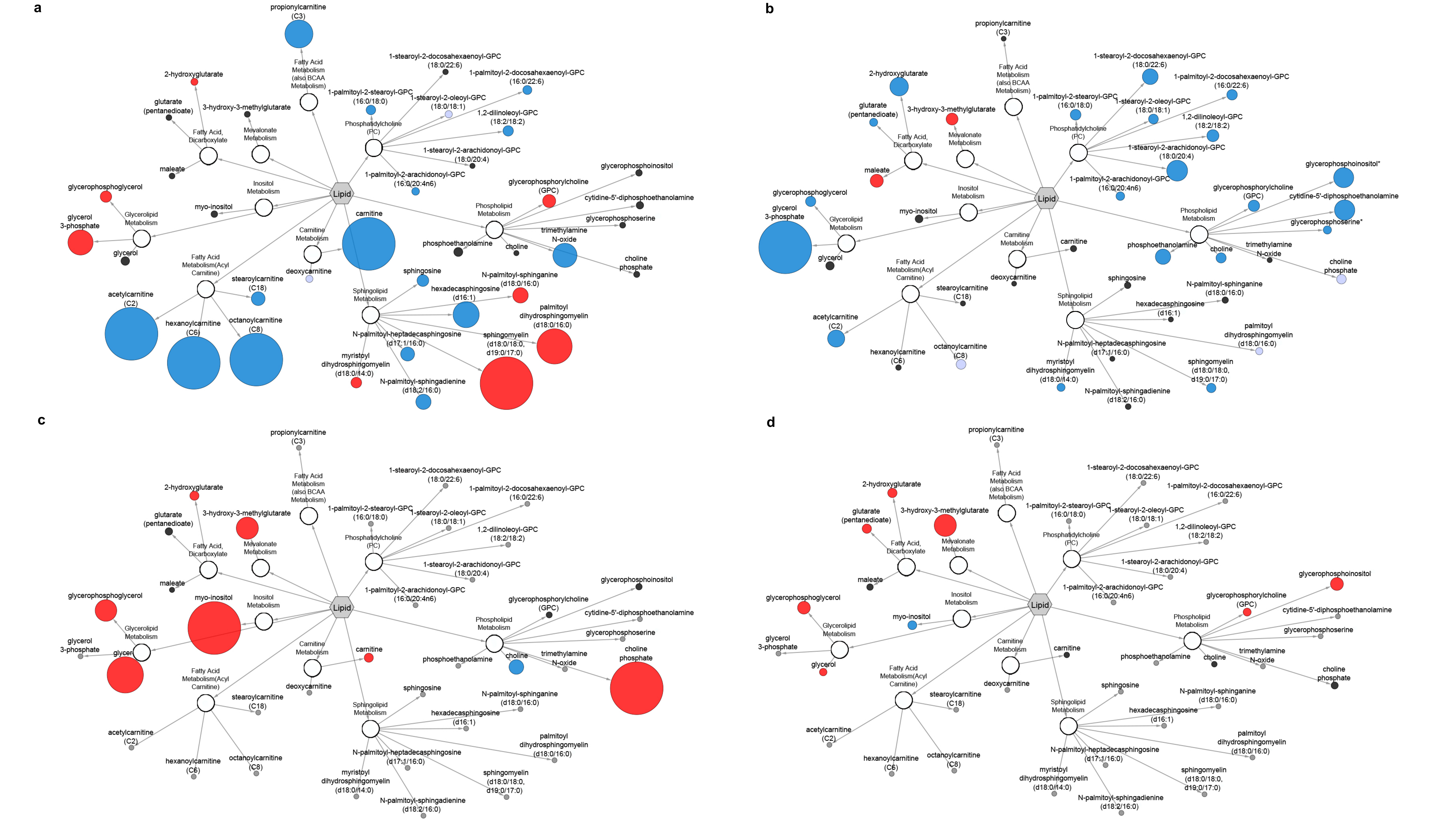

Table S1: List of detected biochemical components in bovine cumulus cells. Fold changes for specific compounds were calculated for cumulus cells matured for 8h versus GV stage cumulus cells (0h); cumulus cells matured for 23h versus GV stage cumulus cells (0h) and cumulus cells matured for 23h versus cumulus cells matured for 8h. In the table, cells were highlighted in red to indicate a significant increase and in blue to indicate a significant decrease. KEGG – Kyoto Encyclopedia of Genes and Genomes Identifier; PUBCHEM – PubChem Compound Identifier; HMDB – Human Metabolon Data Base Identifier.

Table S2: List of biochemical components detected in bovine cumulus-oocyte-complex-conditioned medium. Fold changes for specific compounds were calculated for medium conditioned for 8h versus unconditioned medium (0h); medium conditioned for 23h versus unconditioned medium (0h) and medium conditioned for 23h versus 8h. In the table, cells were highlighted in red to indicate a significant increase or in blue to indicate a significant decrease. KEGG – Kyoto Encyclopedia of Genes and Genomes Identifier; PUBCHEM – PubChem Compound Identifier; HMDB – Human Metabolon Data Base Identifier

Figure S1: Cytoscape specific pathway visualisation of selected components of the amino acid pathway, subpathways are indicated by white circles. a) Cumulus cells at 8h versus 0h; b) cumulus cells at 23h versus 8h; c) maturation medium at 8h versus 0h; d) maturation medium at 23h versus 8h. Each biochemical compound is visualized as a circle, the diameter of which corresponds to the fold change in abundance. Red circles represent a significant increase, blue circles represent a significant decrease, black circles represent biochemical components that were detected but did not change in concentration and grey circles indicate pathway components that were not detected.

Figure S2: Cytoscape specific pathway visualisation of selected components of the carbohydrate pathway, subpathways are indicated by white circles. a) Cumulus cells at 8h versus 0h; b) cumulus cells at 23h versus 8h; c) maturation medium at 8h versus 0h; d) maturation medium at 23h versus 8h. Each biochemical compound is visualized as a circle, the diameter of which corresponds to the fold change in abundance. Red circles represent a significant increase, ligh red circles represent a increasing trend ($0.05<p<0.10$), blue circles represent a significant decrease, light blue circles represent a decreasing trend ($0.05<p<0.10$), black circles represent biochemical components that were detected but did not change in concentration and grey circles indicate pathway components that were not detected.

Figure S3: Cytoscape specific pathway visualisation of selected components of the lipid pathway, subpathways are indicated by the white circles. a) Cumulus cells at 8h versus 0h; b) cumulus cells at 23h versus 8h; c) maturation medium at 8h versus 0h; d) maturation medium at 23h versus 8h. Each component is depicted as a circle, the diameter of which corresponds to the fold change. Red circles represent a significant increase, blue circles represent a significant decrease, light blue circles represent a decreasing trend ($0.05<p<0.10$), black circles represent biochemical compounds that were detected but did not change in abundance and grey circles indicate pathway components that were not detected.

Table S1: List of detected biochemical components in bovine cumulus cells.

Bovine cumulus cells (CCs)

Super Pathway	Sub Pathway	Biochemical Name	KEGG	PUBCHEM	HMDB	Fold of Change 8h vs. 0h	23h vs. 0h	23h vs. 8h
Amino Acid	Glycine, Serine and Threonine Metabolism	glycine	C00037	750	HMDB00123	1.53	2.76	1.80
		N-acetylglycine		10972	HMDB00532	0.85	1.95	2.29
		betaine	C00719	247	HMDB00043	0.06	0.08	1.40
		serine	C00065	5951	HMDB00187	1.18	0.68	0.58
		N-acetylserine		65249	HMDB02931	1.31	1.19	0.91
		threonine	C00188	6288	HMDB00167	1.53	1.82	1.19
		N-acetylthreonine		152204		1.73	1.89	1.09
	Alanine and Aspartate Metabolism	alanine	C00041	5950	HMDB00161	1.14	1.48	1.29
		N-acetylalanine	C02847	88064	HMDB00766	1.17	0.73	0.62
		aspartate	C00049	5960	HMDB00191	1.89	9.29	4.92
		N-acetylaspartate (NAA)	C01042	65065	HMDB00812	1.36	6.87	5.04
		asparagine	C00152	6267	HMDB00168	0.78	0.54	0.70
	Glutamate Metabolism	glutamate	C00025	611	HMDB00148	1.48	1.60	1.08
		glutamine	C00064	5961	HMDB00641	3.03	3.02	1.00
		N-acetylglutamate	C00624	70914	HMDB01138	1.98	1.66	0.84
		N-acetylglutamine	C02716	182230	HMDB06029	4.72	2.71	0.57
		pyroglutamine		134508		0.37	1.72	4.62
		N-acetyl-aspartyl-glutamate (NAAG)	C12270	5255	HMDB01067	0.72	0.72	0.99
		beta-citrylglutamate	C20775	72715786		1.07	0.06	0.06
	Histidine Metabolism	histidine	C00135	6274	HMDB00177	1.59	2.23	1.40
		carnosine	C00386	439224	HMDB00033	0.64	0.64	1.00
		4-imidazoleacetate	C02835	96215	HMDB02024	3.09	8.14	2.63
	Lysine Metabolism	lysine	C00047	5962	HMDB00182	1.48	2.81	1.90
		N6,N6,N6-trimethyllysine	C03793	440120	HMDB01325	0.53	0.96	1.80
		5-(galactosylhydroxy)-L-lysine				1.17	0.65	0.55
		2-aminoadipate	C00956	469	HMDB00510	2.21	2.79	1.26
		N-trimethyl 5-aminovalerate				0.08	0.04	0.53
	Phenylalanine Metabolism	phenylalanine	C00079	6140	HMDB00159	1.65	3.17	1.93
	Tyrosine Metabolism	tyrosine	C00082	6057	HMDB00158	1.66	2.82	1.70
		3-(4-hydroxyphenyl)lactate	C03672	9378	HMDB00755	1.59	1.80	1.13
		phenol sulfate	C02180	74426	HMDB60015	2.61	4.25	1.63
	Tryptophan Metabolism	tryptophan	C00078	6305	HMDB00929	2.38	5.06	2.13
	Leucine, Isoleucine and Valine Metabolism	leucine	C00123	6106	HMDB00687	1.78	3.08	1.73
		4-methyl-2-oxopentanoate	C00233	70	HMDB00695	1.03	1.82	1.77
		beta-hydroxyisovalerate		69362	HMDB00754	1.16	1.18	1.01
		isoleucine	C00407	6306	HMDB00172	2.48	5.40	2.17
		3-methyl-2-oxovalerate	C00671	47	HMDB03736	1.41	2.74	1.95
		methylsuccinate		10349	HMDB01844	1.31	1.91	1.46
		valine	C00183	6287	HMDB00883	1.45	2.68	1.85
		3-methyl-2-oxobutyrate	C00141	49	HMDB00019	0.72	1.10	1.54
	Methionine, Cysteine, SAM and Taurine Metabolism	methionine	C00073	6137	HMDB00696	1.59	2.46	1.55
		N-acetylmethionine	C02712	448580	HMDB11745	0.98	0.26	0.27
		methionine sulfoxide	C02989	158980	HMDB02005	1.63	4.85	2.97
		S-adenosylmethionine (SAM)	C00019	34756	HMDB01185	0.74	1.00	1.35
		S-adenosylhomocysteine (SAH)	C00021	439155	HMDB00939	0.99	0.97	0.98
		cystathionine	C02291	439258	HMDB00099	0.97	0.52	0.54
		cysteine	C00097	5862	HMDB00574	1.45	1.96	1.35
		N-acetylcysteine	C06809	12035	HMDB01890	2.09	1.00	0.48
		hypotaurine	C00519	107812	HMDB00965	3.67	5.57	1.52
		taurine	C00245	1123	HMDB00251	0.04	0.21	4.80
	Urea cycle; Arginine and Proline Metabolism	arginine	C00062	232	HMDB00517	1.20	1.69	1.41
		argininosuccinate	C03406	828	HMDB00052	1.80	0.78	0.43
		urea	C00086	1176	HMDB00294	0.80	1.97	2.47
		ornithine	C00077	6262	HMDB03374	3.56	5.14	1.44
		proline	C00148	145742	HMDB00162	1.85	2.15	1.16
		dimethylarginine (SDMA + ADMA)	C03626	123831	HMDB01539	0.57	0.66	1.17
		trans-4-hydroxyproline	C01157	5810	HMDB00725	2.89	4.44	1.54
	Creatine Metabolism	guanidinoacetate	C00581	763	HMDB00128	0.46	0.61	1.33
		creatine	C00300	586	HMDB00064	0.17	0.06	0.37
		creatinine	C00791	588	HMDB00562	0.28	0.08	0.28
		creatine phosphate	C02305	587	HMDB01511	1.03	0.48	0.46
	Polyamine Metabolism	putrescine	C00134	1045	HMDB01414	0.22	0.21	0.98
		spermidine	C00315	1102	HMDB01257	1.71	1.31	0.76
		N1,N12-diacetylspermine	C03413	132680	HMDB02172	1.00	1.87	1.87
		5-methylthioadenosine (MTA)	C00170	439176	HMDB01173	0.83	0.51	0.62
		N-acetylputrescine	C02714	122356	HMDB02064	0.59	0.80	1.35
		(N(1) + N(8))-acetylspermidine				1.00	3.46	3.46
	Guanidino and Acetamido Metabolism	4-guanidinobutanoate	C01035	500	HMDB03464	1.16	1.35	1.16
	Glutathione Metabolism	glutathione, reduced (GSH)	C00051	124886	HMDB00125	1.49	0.43	0.29
		glutathione, oxidized (GSSG)	C00127	65359	HMDB03337	1.21	1.24	1.02
		S-methylglutathione	C11347	3605667		0.88	0.68	0.77
		5-oxoproline	C01879	7405	HMDB00267	1.43	2.55	1.78
		2-hydroxybutyrate/2-hydroxyisobutyrate				1.21	1.93	1.59
		ophthalmate		7018721	HMDB05765	0.06	0.05	0.71
		S-nitrosoglutathione (GSNO)		3514	HMDB04645	1.97	0.92	0.47
Peptide	Gamma-glutamyl Amino Acid	gamma-glutamylglutamine	C05283	150914	HMDB11738	2.00	2.61	1.30
		gamma-glutamylthreonine		76078708	HMDB29159	3.82	0.86	0.22
	Dipeptide	leucylglycine		79070	HMDB28929	1.00	4.00	4.00
		phenylalanylglycine		98207	HMDB28995	0.93	0.44	0.47
	Acetylated Peptides	phenylacetylglycine	C05598	68144	HMDB00821	0.96	1.77	1.86
Carbohydrate	Glycolysis, Gluconeogenesis, and Pyruvate Metabolism	glucose	C00031	79025	HMDB00122	1.24	0.49	0.40
		3-phosphoglycerate	C00597	724	HMDB00807	1.12	0.02	0.02
		phosphoenolpyruvate (PEP)	C00074	1005	HMDB00263	1.55	0.03	0.02
		pyruvate	C00022	1060	HMDB00243	1.23	2.15	1.75
		lactate	C00186	612	HMDB00190	1.26	2.41	1.91
		glycerate	C00258	752	HMDB00139	1.06	0.26	0.25
	Pentose Phosphate Pathway	sedoheptulose-7-phosphate	C05382	616	HMDB01068	3.13	1.01	0.32
	Pentose Metabolism	ribose	C00121	5779	HMDB00283	0.79	0.20	0.25
		ribonate	C01685	5460677	HMDB00867	1.42	1.61	1.13
		arabonate/xylonate				1.31	2.02	1.55
		sedoheptulose		5459879	HMDB03219	1.01	1.13	1.12
	Glycogen Metabolism	maltotetraose	C02052	446495	HMDB01296	0.27	0.26	0.95
		maltotriose	C01835	439586	HMDB01262	0.27	0.09	0.33
		maltose	C00208	10991489	HMDB00163	0.28	0.37	1.32
	Disaccharides and Oligosaccharides	lactose	C00243	84571	HMDB00186	0.39	0.08	0.21
	Fructose, Mannose and Galactose Metabolism	fructose	C00095	5984	HMDB00660	3.71	7.79	2.10
		mannitol/sorbitol	C00794	5780	HMDB00247	1.13	0.50	0.44
		mannose	C00159	18950	HMDB00169	1.21	1.03	0.85
	Nucleotide Sugar	UDP-glucose	C00029	8629	HMDB00286	10.53	3.70	0.35
		UDP-galactose	C00052	18068	HMDB00302	8.74	1.72	0.20
		UDP-glucuronate	C00167	17473	HMDB00935	1.38	1.03	0.74
		UDP-N-acetylglucosamine/galactosamine				0.69	5.58	8.09
		cytidine 5'-monophospho-N-acetylneuraminic acid	C00128	448209	HMDB01176	2.86	1.07	0.37
	Aminosugar Metabolism	glucuronate	C00191	444791	HMDB00127	0.88	0.59	0.67
		N-acetylglucosamine 6-phosphate	C00357	439219	HMDB02817	0.63	0.53	0.84
		N-acetyl-glucosamine 1-phosphate	C04256	440364	HMDB01367	0.50	3.95	7.86
		N-acetylneuraminate	C00270	439197	HMDB00230	0.54	0.24	0.44
		erythronate		2781043	HMDB00613	1.85	2.07	1.12

		N-acetylglucosamine/N-acetylgalactosamine		24139	HMDB00215	0.58	1.02	1.77
Energy	TCA Cycle	citrate	C00158	311	HMDB00094	1.06	0.12	0.11
		aconitate [cis or trans]				1.18	0.29	0.24
		alpha-ketoglutarate	C00026	51	HMDB00208	1.16	0.60	0.52
		succinate	C00042	1110	HMDB00254	1.40	1.03	0.74
		fumarate	C00122	444972	HMDB00134	1.81	0.67	0.37
		malate	C00149	525	HMDB00156	1.61	0.32	0.20
	Oxidative Phosphorylation	acetylphosphate	C00227	186	HMDB01494	0.74	0.10	0.13
		phosphate	C00009	1061	HMDB01429	1.19	1.12	0.94
Lipid	Medium Chain Fatty Acid	caprylate (8:0)	C06423	379	HMDB00482	1.80	4.41	2.44
	Long Chain Fatty Acid	myristoleate (14:1n5)	C08322	5281119	HMDB02000	0.80	0.48	0.60
		palmitate (16:0)	C00249	985	HMDB00220	1.46	0.96	0.66
		palmitoleate (16:1n7)	C08362	445638	HMDB03229	0.80	0.40	0.50
		10-heptadecenoate (17:1n7)		5312435	HMDB60038	0.97	0.64	0.66
		oleate/vaccenate (18:1)				0.82	0.67	0.82
		10-nonadecenoate (19:1n9)		5312513	HMDB13622	1.17	0.88	0.76
		eicosenoate (20:1)	C16526	5282768	HMDB02231	1.22	1.19	0.97
		erucate (22:1n9)	C08316	5281116	HMDB02068	1.32	1.75	1.33
		nervonate (24:1n9)	C08323	5281120	HMDB02368	0.99	2.28	2.30
	Polyunsaturated Fatty Acid (n3 and n6)	eicosapentaenoate (EPA; 20:5n3)	C06428	446284	HMDB01999	0.58	0.21	0.36
		docosapentaenoate (n3 DPA; 22:5n3)	C16513	6441454	HMDB06528	1.04	0.91	0.87
		docosahexaenoate (DHA; 22:6n3)	C06429	445580	HMDB02183	0.36	0.28	0.78
		linoleate (18:2n6)	C01595	5280450	HMDB00673	1.01	0.66	0.66
		linolenate [alpha or gamma; (18:3n3 or 6)]	C06426	5280934	HMDB03073	0.58	0.23	0.39
		dihomo-linolenate (20:3n3 or n6)	C03242	5280581	HMDB02925	0.96	0.72	0.75
		arachidonate (20:4n6)	C00219	444899	HMDB01043	0.86	0.51	0.59
		docosadienoate (22:2n6)	C16533	5282807	HMDB61714	1.05	0.95	0.91
		dihomo-linoleate (20:2n6)	C16525	6439848	HMDB05060	1.23	0.89	0.72
		mead acid (20:3n9)		5312531	HMDB10378	0.94	0.63	0.66
	Fatty Acid, Dicarboxylate	glutarate (pentanedioate)	C00489	743	HMDB00661	1.16	0.73	0.63
		2-hydroxyglutarate	C02630	43	HMDB00606	1.38	0.39	0.28
		maleate	C01384	444266	HMDB00176	1.17	2.93	2.51
	Fatty Acid Metabolism (also BCAA Metabolism)	propionylcarnitine (C3)	C03017	107738	HMDB00824	0.19	0.19	1.00
		methylmalonate (MMA)	C02170	487	HMDB00202	0.95	0.88	0.93
	Fatty Acid Metabolism(Acyl Carnitine)	acetylcarnitine (C2)	C02571	1	HMDB00201	0.07	0.02	0.31
		3-hydroxybutyrylcarnitine (1)		53481617	HMDB13127	0.86	0.86	1.00
		3-hydroxybutyrylcarnitine (2)			HMDB13127	0.72	0.72	1.00
		hexanoylcarnitine (C6)		6426853	HMDB00705	0.08	0.07	0.90
		octanoylcarnitine (C8)	C02838	123701	HMDB00791	0.06	0.03	0.53
		stearoylcarnitine (C18)		6426855	HMDB00848	0.38	0.39	1.04
	Carnitine Metabolism	deoxycarnitine	C01181	134	HMDB01161	0.72	0.72	1.00
		carnitine	C00318	10917	HMDB00062	0.06	0.08	1.25
	Fatty Acid, Monohydroxy	2-hydroxypalmitate		92836	HMDB31057	1.00	0.44	0.44
		3-hydroxyhexanoate		151492		1.14	1.24	1.09
		3-hydroxyoctanoate		26613	HMDB01954	1.18	1.00	0.85
		3-hydroxylaurate		94216	HMDB00387	1.02	1.29	1.26
		13-HODE + 9-HODE		43013		2.21	1.19	0.54
	Endocannabinoid	oleoyl ethanolamide		5283454	HMDB02088	0.27	0.15	0.55
	Inositol Metabolism	myo-inositol	C00137	892	HMDB00211	0.76	0.50	0.65
	Phospholipid Metabolism	choline	C00114	305	HMDB00097	1.05	0.54	0.51
		choline phosphate	C00588	1014	HMDB01565	0.82	0.44	0.54
		glycerophosphorylcholine (GPC)	C00670	71920	HMDB00086	2.52	1.14	0.45
		phosphoethanolamine	C00346	1015	HMDB00224	0.57	0.19	0.34
		cytidine-5'-diphosphoethanolamine	C00570	123727	HMDB01564	0.68	0.18	0.26
		glycerophosphoethanolamine	C01233	123874	HMDB00114	1.98	0.95	0.48
		glycerophosphoserine		3081457		1.18	0.74	0.63
		glycerophosphoinositol		167572		1.30	0.34	0.26
		trimethylamine N-oxide	C01104	1145	HMDB00925	0.22	0.22	1.03
	Phosphatidylcholine (PC)	1-myristoyl-2-palmitoyl-GPC (14:0/16:0)		129657	HMDB07869	0.84	0.77	0.91
		1,2-dipalmitoyl-GPC (16:0/16:0)	D03585	452110	HMDB00564	0.66	0.60	0.91
		1-palmitoyl-2-palmitoleoyl-GPC (16:0/16:1)			HMDB07969	0.80	0.60	0.75
		1-palmitoyl-2-stearoyl-GPC (16:0/18:0)			HMDB07970	0.54	0.26	0.48
		1-palmitoyl-2-oleoyl-GPC (16:0/18:1)		6436017	HMDB07972	0.82	0.68	0.83
		1-palmitoyl-2-linoleoyl-GPC (16:0/18:2)		5287971	HMDB07973	0.75	0.53	0.70
		1-palmitoyl-2-alpha-linolenoyl-GPC (16:0/18:3n3)			HMDB07975	0.57	0.22	0.40
		1-palmitoleoyl-2-linoleoyl-GPC (16:1/18:2)			HMDB08006	0.51	0.27	0.53
		1-palmitoleoyl-2-linolenoyl-GPC (16:1/18:3)			HMDB08008	0.59	0.24	0.40
		1-palmitoyl-2-arachidonoyl-GPC (16:0/20:4n6)		10747814	HMDB07982	0.67	0.40	0.60
		1-palmitoyl-2-docosahexaenoyl-GPC (16:0/22:6)		6441886	HMDB07991	0.57	0.28	0.49
		1-stearoyl-2-oleoyl-GPC (18:0/18:1)			HMDB08038	0.70	0.37	0.53
		1,2-dioleoyl-GPC (18:1/18:1)		10350317		0.99	0.77	0.78
		1-oleoyl-2-linoleoyl-GPC (18:1/18:2)				0.72	0.47	0.66
		1,2-dilinoleoyl-GPC (18:2/18:2)		5288075	HMDB08138	0.50	0.21	0.43
		1-linoleoyl-2-linolenoyl-GPC (18:2/18:3)			HMDB08141	0.41	0.20	0.49
		1-stearoyl-2-arachidonoyl-GPC (18:0/20:4)		16219824	HMDB08048	1.06	0.27	0.25
		1-stearoyl-2-docosahexaenoyl-GPC (18:0/22:6)			HMDB08057	0.90	0.30	0.33
		1-oleoyl-2-docosahexaenoyl-GPC (18:1/22:6)			HMDB08123	0.64	0.29	0.45
	Phosphatidylethanolamine (PE)	1,2-dipalmitoyl-GPE (16:0/16:0)		445468	HMDB08923	0.59	0.36	0.62
		1-palmitoyl-2-stearoyl-GPE (16:0/18:0)		5326793	HMDB08925	0.83	0.44	0.54
		1-palmitoyl-2-oleoyl-GPE (16:0/18:1)		5283496	HMDB05320	0.73	0.37	0.52
		1-palmitoyl-2-linoleoyl-GPE (16:0/18:2)		9546747	HMDB05322	0.77	0.40	0.52
		1-palmitoleoyl-2-oleoyl-GPE (16:1/18:1)				0.60	0.24	0.41
		1-palmitoyl-2-arachidonoyl-GPE (16:0/20:4)		9546800	HMDB05323	0.56	0.30	0.53
		1-palmitoyl-2-docosahexaenoyl-GPE (16:0/22:6)		9546799	HMDB05324	0.76	0.42	0.55
		1-stearoyl-2-oleoyl-GPE (18:0/18:1)			HMDB08993	0.68	0.40	0.59
		1,2-dioleoyl-GPE (18:1/18:1)		9546757		0.71	0.38	0.54
		1-oleoyl-2-linoleoyl-GPE (18:1/18:2)		9546753	HMDB05349	0.67	0.37	0.55
		1-stearoyl-2-arachidonoyl-GPE (18:0/20:4)		5289133	HMDB09003	0.72	0.41	0.57
		1-oleoyl-2-arachidonoyl-GPE (18:1/20:4)			HMDB09069	0.63	0.35	0.56
		1-stearoyl-2-docosahexaenoyl-GPE (18:0/22:6)		9546798	HMDB05334	0.94	0.57	0.61
		1-oleoyl-2-docosahexaenoyl-GPE (18:1/22:6)				0.80	0.43	0.54
	Phosphatidylserine (PS)	1-palmitoyl-2-oleoyl-GPS (16:0/18:1)	C13880	5283499	HMDB12357	0.78	0.48	0.62
		1-stearoyl-2-oleoyl-GPS (18:0/18:1)		9547087	HMDB10163	0.74	0.33	0.44
	Phosphatidylinositol (PI)	1-palmitoyl-2-arachidonoyl-GPI (16:0/20:4)			HMDB09789	0.58	0.40	0.69
		1-stearoyl-2-oleoyl-GPI (18:0/18:1)				1.53	0.74	0.49
		1-stearoyl-2-arachidonoyl-GPI (18:0/20:4)			HMDB09815	0.56	0.44	0.78
		1-oleoyl-2-arachidonoyl-GPI (18:1/20:4)			HMDB09844	0.97	0.58	0.60
	Lysophospholipid	1-palmitoyl-GPC (16:0)		86554	HMDB10382	1.40	2.05	1.47
		2-palmitoyl-GPC (16:0)		15061532	HMDB61702	0.83	0.73	0.88
		1-palmitoleoyl-GPC (16:1)		24779461	HMDB10383	1.59	4.04	2.54
		1-stearoyl-GPC (18:0)		497299	HMDB10384	1.13	1.53	1.36
		1-oleoyl-GPC (18:1)		16081932	HMDB02815	1.61	2.64	1.64
		1-linoleoyl-GPC (18:2)	C04100	11988421	HMDB10386	1.60	9.07	5.66
		1-palmitoyl-GPE (16:0)		9547069	HMDB11503	1.14	1.13	1.00
		1-stearoyl-GPE (18:0)		9547068	HMDB11130	1.15	0.97	0.84
		1-oleoyl-GPE (18:1)		9547071	HMDB11506	0.93	1.35	1.45
		1-oleoyl-GPG (18:1)				1.25	4.16	3.32
		1-stearoyl-GPI (18:0)			HMDB61696	1.23	1.39	1.13
		1-oleoyl-GPI (18:1)				2.35	2.26	0.96
		1-arachidonoyl-GPI (20:4)			HMDB61690	1.60	1.47	0.92
	Plasmalogen	1-(1-enyl-palmitoyl)-2-oleoyl-GPE (P-16:0/18:1)			HMDB11342	0.80	0.56	0.70
		1-(1-enyl-palmitoyl)-2-linoleoyl-GPE (P-16:0/18:2)			HMDB11343	0.91	0.64	0.70
		1-(1-enyl-palmitoyl)-2-palmitoyl-GPC (P-16:0/16:0)		11146967	HMDB11206	0.72	0.52	0.73
		1-(1-enyl-palmitoyl)-2-palmitoleoyl-GPC (P-16:0/16:1)			HMDB11207	0.83	0.71	0.85
		1-(1-enyl-palmitoyl)-2-arachidonoyl-GPE (P-16:0/20:4)			HMDB11352	0.77	0.58	0.76
		1-(1-enyl-palmitoyl)-2-oleoyl-GPC (P-16:0/18:1)				0.79	0.81	1.02
		1-(1-enyl-stearoyl)-2-oleoyl-GPE (P-18:0/18:1)			HMDB11375	1.16	0.39	0.34

		1-(1-enyl-palmitoyl)-2-arachidonoyl-GPC (P-16:0/20:4)			HMDB11220	1.18	1.73	1.46
		1-(1-enyl-palmitoyl)-2-linoleoyl-GPC (P-16:0/18:2)			HMDB11211	0.71	0.68	0.96
		1-(1-enyl-stearoyl)-2-arachidonoyl-GPE (P-18:0/20:4)		9547058	HMDB05779	0.70	0.50	0.72
	Lysoplasmalogen	1-(1-enyl-oleoyl)-2-oleoyl-GPE (P-18:1/18:1)			HMDB11441	0.81	0.59	0.73
	Glycerolipid Metabolism	glycerol	C00116	753	HMDB00131	0.60	0.98	1.65
		glycerol 3-phosphate	C00093	754	HMDB00126	4.79	0.30	0.06
		glycerophosphoglycerol	C03274	439964		2.20	1.11	0.51
	Monoacylglycerol	1-pentadecanoylglycerol (15:0)		190750		1.22	1.13	0.93
		1-palmitoylglycerol (16:0)		14900	HMDB31074	1.56	2.30	1.47
		1-palmitoleoylglycerol (16:1)			HMDB11565	2.51	3.92	1.56
		1-oleoylglycerol (18:1)		5283468	HMDB11567	2.97	6.85	2.31
		1-linoleoylglycerol (18:2)		5283469		0.85	1.70	1.99
		2-myristoylglycerol (14:0)		137938	HMDB11530	1.18	0.80	0.68
		2-palmitoylglycerol (16:0)		123409	HMDB11533	0.85	1.32	1.56
		2-oleoylglycerol (18:1)		5319879	HMDB11537	5.47	0.95	0.17
		2-linoleoylglycerol (18:2)		5365676	HMDB11538	1.85	1.34	0.72
	Diacylglycerol	diacylglycerol (12:0/18:1, 14:0/16:1, 16:0/14:1) [2]				0.76	0.45	0.59
		diacylglycerol (14:0/18:1, 16:0/16:1) [1]				1.03	0.82	0.79
		diacylglycerol (14:0/18:1, 16:0/16:1) [2]				0.82	0.41	0.50
		diacylglycerol (16:1/18:2 [2], 16:0/18:3 [1])				0.64	0.34	0.53
		palmitoyl-myristoyl-glycerol (16:0/14:0) [2]			HMDB07095	1.08	0.55	0.50
		palmitoyl-palmitoyl-glycerol (16:0/16:0) [1]			HMDB07098	1.37	0.96	0.70
		palmitoyl-palmitoyl-glycerol (16:0/16:0) [2]			HMDB07098	1.02	0.44	0.43
		palmitoyl-oleoyl-glycerol (16:0/18:1) [1]	C13861		HMDB07102	1.18	0.94	0.80
		palmitoyl-oleoyl-glycerol (16:0/18:1) [2]	C13861		HMDB07102	0.83	0.44	0.53
		palmitoyl-linoleoyl-glycerol (16:0/18:2) [2]			HMDB07103	0.70	0.33	0.48
		palmitoleoyl-oleoyl-glycerol (16:1/18:1) [2]				0.77	0.43	0.56
		palmitoleoyl-linoleoyl-glycerol (16:1/18:2) [1]			HMDB07132	0.62	0.47	0.77
		palmitoyl-dihomo-linolenoyl-glycerol (16:0/20:3n3 or 6) [2]				0.31	0.08	0.26
		palmitoyl-arachidonoyl-glycerol (16:0/20:4) [1]			HMDB07112	1.35	0.80	0.60
		palmitoyl-arachidonoyl-glycerol (16:0/20:4) [2]			HMDB07112	0.87	0.40	0.45
		palmitoyl-docosahexaenoyl-glycerol (16:0/22:6) [1]			HMDB07121	0.78	0.30	0.38
		oleoyl-oleoyl-glycerol (18:1/18:1) [1]			HMDB07218	0.96	0.89	0.92
		oleoyl-oleoyl-glycerol (18:1/18:1) [2]			HMDB07218	0.71	0.45	0.63
		oleoyl-linoleoyl-glycerol (18:1/18:2) [2]			HMDB07219	0.69	0.40	0.58
		oleoyl-linolenoyl-glycerol (18:1/18:3) [2]			HMDB07220	0.67	0.35	0.52
		linoleoyl-linoleoyl-glycerol (18:2/18:2) [1]			HMDB07248	0.82	0.89	1.08
		stearoyl-arachidonoyl-glycerol (18:0/20:4) [1]				0.84	0.78	0.93
		stearoyl-arachidonoyl-glycerol (18:0/20:4) [2]				0.61	0.42	0.70
		oleoyl-arachidonoyl-glycerol (18:1/20:4) [1]			HMDB07228	0.93	0.71	0.77
		oleoyl-arachidonoyl-glycerol (18:1/20:4) [2]			HMDB07228	0.87	0.55	0.64
		linoleoyl-arachidonoyl-glycerol (18:2/20:4) [1]			HMDB07257	1.00	0.31	0.31
		linoleoyl-arachidonoyl-glycerol (18:2/20:4) [2]			HMDB07257	0.70	0.12	0.18
		stearoyl-docosahexaenoyl-glycerol (18:0/22:6) [1]				0.52	0.42	0.80
		stearoyl-docosahexaenoyl-glycerol (18:0/22:6) [2]				0.67	0.55	0.82
	Sphingolipid Metabolism	sphinganine	C00836	3126	HMDB00269	1.67	1.87	1.13
		N-palmitoyl-sphinganine (d18:0/16:0)		5283572	HMDB11760	2.96	3.74	1.26
		N-palmitoyl-sphingadienine (d18:2/16:0)				0.34	0.39	1.15
		myristoyl dihydrosphingomyelin (d18:0/14:0)			HMDB12085	2.06	1.31	0.64
		palmitoyl dihydrosphingomyelin (d18:0/16:0)		9939965		6.69	4.76	0.71
		palmitoyl sphingomyelin (d18:1/16:0)		9939941		1.14	0.91	0.80
		stearoyl sphingomyelin (d18:1/18:0)	C00550	6453725	HMDB01348	1.15	0.93	0.81
		behenoyl sphingomyelin (d18:1/22:0)			HMDB12103	1.01	0.66	0.66
		tricosanoyl sphingomyelin (d18:1/23:0)			HMDB12105	0.68	0.36	0.53
		lignoceroyl sphingomyelin (d18:1/24:0)				0.94	0.57	0.61
		sphingomyelin (d18:1/14:0, d16:1/16:0)		11433862	HMDB12097	0.82	0.43	0.52
		sphingomyelin (d17:1/16:0, d18:1/15:0, d16:1/17:0)				0.75	0.34	0.45
		sphingomyelin (d18:2/16:0, d18:1/16:1)				0.86	0.37	0.43
		sphingomyelin (d18:1/17:0, d17:1/18:0, d19:1/16:0)				0.95	0.49	0.52
		sphingomyelin (d18:1/18:1, d18:2/18:0)		6443882	HMDB12101	1.18	0.73	0.62
		sphingomyelin (d18:1/20:0, d16:1/22:0)			HMDB12102	1.01	0.54	0.54
		sphingomyelin (d18:1/22:1, d18:2/22:0, d16:1/24:1)			HMDB12104	0.96	0.50	0.53
		sphingomyelin (d18:2/23:0, d18:1/23:1, d17:1/24:1)				0.77	0.36	0.47
		sphingomyelin (d18:1/24:1, d18:2/24:0)			HMDB12107	0.97	0.56	0.57
		sphingomyelin (d18:2/24:1, d18:1/24:2)				0.93	0.43	0.47
		sphingosine	C00319	5353955	HMDB00252	0.44	0.57	1.29
		sphingomyelin (d18:2/23:1)				0.76	0.26	0.34
		sphingomyelin (d18:2/24:2)				1.61	0.77	0.48
		sphingomyelin (d18:1/22:2, d18:2/22:1, d16:1/24:2)				1.21	1.00	0.82
		sphingomyelin (d18:0/18:0, d19:0/17:0)			HMDB12087	9.99	5.51	0.55
		hexadecasphingosine (d16:1)				0.20	0.21	1.03
		N-palmitoyl-heptadecasphingosine (d17:1/16:0)				0.37	0.38	1.01
	Ceramides	N-palmitoyl-sphingosine (d18:1/16:0)		5283564	HMDB04949	0.46	0.55	1.19
		N-stearoyl-sphingosine (d18:1/18:0)		5283565	HMDB04950	0.40	0.43	1.07
		ceramide (d16:1/24:1, d18:1/22:1)				0.47	0.53	1.14
		ceramide (d18:1/17:0, d17:1/18:0)				0.34	0.36	1.08
		ceramide (d18:2/24:1, d18:1/24:2)				0.48	0.49	1.02
		glycosyl-N-palmitoyl-sphingosine (d18:1/16:0)				0.52	0.27	0.51
		glycosyl-N-stearoyl-sphingosine (d18:1/18:0)				0.64	0.64	1.00
		lactosyl-N-palmitoyl-sphingosine (d18:1/16:0)				1.00	1.00	1.00
	Mevalonate Metabolism	3-hydroxy-3-methylglutarate	C03761	1662	HMDB00355	0.84	1.93	2.29
	Sterol	cholesterol	C00187	11025495	HMDB00067	0.81	0.58	0.72
	Primary Bile Acid Metabolism	cholate	C00695	221493	HMDB00619	0.76	0.97	1.28
		glycocholate	C01921	10140	HMDB00138	0.79	1.01	1.28
		taurocholate	C05122	6675	HMDB00036	0.91	1.22	1.33
	Secondary Bile Acid Metabolism	glycodeoxycholate	C05464	3035026	HMDB00631	1.10	1.36	1.24
		taurodeoxycholate	C05463	2733768	HMDB00896	1.19	1.48	1.25
Nucleotide	Purine Metabolism, (Hypo)Xanthine/Inosine containing	inosine	C00294	6021	HMDB00195	1.13	0.70	0.62
		hypoxanthine	C00262	790	HMDB00157	0.95	0.55	0.57
		xanthine	C00385	1188	HMDB00292	0.82	0.81	0.99
		xanthosine	C01762	64959	HMDB00299	0.47	0.34	0.73
		2'-deoxyinosine	C05512	65058	HMDB00071	1.03	0.47	0.45
		urate	C00366	1175	HMDB00289	0.21	0.20	0.98
		allantoin	C02350	204	HMDB00462	0.02	0.05	2.48
	Purine Metabolism, Adenine containing	adenosine 5'-monophosphate (AMP)	C00020	6083	HMDB00045	1.23	0.43	0.35
		adenosine 3'-monophosphate (3'-AMP)	C01367	41211	HMDB03540	1.27	0.22	0.17
		adenosine 2'-monophosphate (2'-AMP)	C00946	94136	HMDB11617	0.64	0.17	0.26
		adenosine 3',5'-cyclic monophosphate (cAMP)	C00575	6076	HMDB00058	3.34	1.07	0.32
		adenosine	C00212	60961	HMDB00050	2.46	6.34	2.58
		adenine	C00147	190	HMDB00034	0.60	1.70	2.81
	Purine Metabolism, Guanine containing	guanosine	C00387	6802	HMDB00133	1.49	0.85	0.57
		guanine	C00242	764	HMDB00132	1.19	0.51	0.43
		2'-deoxyguanosine	C00330	187790	HMDB00085	0.49	0.27	0.55
	Pyrimidine Metabolism, Orotate containing	orotate	C00295	967	HMDB00226	1.92	3.05	1.59
		orotidine		92751	HMDB00788	2.89	1.27	0.44
	Pyrimidine Metabolism, Uracil containing	uridine	C00299	6029	HMDB00296	1.17	0.85	0.73
		uracil	C00106	1174	HMDB00300	0.89	0.54	0.60
		pseudouridine	C02067	15047	HMDB00767	0.98	2.65	2.70
		2'-deoxyuridine	C00526	13712	HMDB00012	0.96	0.96	1.01
		3-ureidopropionate	C02642	111	HMDB00026	0.02	0.06	2.57
		beta-alanine	C00099	239	HMDB00056	0.57	0.81	1.44
		N-acetyl-beta-alanine	C01073	76406		1.00	1.45	1.45
	Pyrimidine Metabolism, Cytidine containing	cytidine 5'-monophosphate (5'-CMP)	C00055	6131	HMDB00095	1.38	0.82	0.60
		cytidine	C00475	6175	HMDB00089	0.66	0.36	0.55
		cytosine	C00380	597	HMDB00630	0.58	0.36	0.62
		2'-deoxycytidine	C00881	13711	HMDB00014	0.38	0.46	1.21

	Pyrimidine Metabolism, Thymine containing	thymidine	C00214	5789	HMDB00273	0.46	0.25	0.53
	Purine and Pyrimidine Metabolism	methylphosphate		13130	HMDB61711	1.38	0.24	0.18
Cofactors and Vitamins	Nicotinate and Nicotinamide Metabolism	nicotinamide	C00153	936	HMDB01406	1.22	0.36	0.30
		nicotinamide ribonucleotide (NMN)	C00455	14180	HMDB00229	1.91	1.50	0.78
		nicotinamide riboside	C03150	439924	HMDB00855	0.93	0.39	0.42
		nicotinamide adenine dinucleotide (NAD+)	C00003	5893	HMDB00902	3.41	2.12	0.62
	Riboflavin Metabolism	flavin adenine dinucleotide (FAD)	C00016	643975	HMDB01248	0.95	0.42	0.44
	Pantothenate and CoA Metabolism	pantothenate	C00864	6613	HMDB00210	0.38	0.12	0.32
	Ascorbate and Aldarate Metabolism	threonate	C01620	151152	HMDB00943	0.86	1.68	1.95
		gulonate	C00257	9794176	HMDB03290	0.99	0.69	0.70
	Tocopherol Metabolism	alpha-tocopherol	C02477	14985	HMDB01893	1.12	0.67	0.60
	Vitamin A Metabolism	retinol (Vitamin A)	C00473	445354	HMDB00305	0.43	0.29	0.69
	Vitamin B6 Metabolism	pyridoxal	C00250	1050	HMDB01545	2.30	1.84	0.80
Xenobiotics	Benzoate Metabolism	hippurate	C01586	464	HMDB00714	0.04	0.06	1.66
		benzoate	C00180	243	HMDB01870	1.08	1.36	1.27
		p-cresol sulfate		4615423	HMDB11635	0.60	1.74	2.91
	Food Component/Plant	gluconate	C00257	10690	HMDB00625	1.30	0.88	0.67
		beta-guanidinopropanoate	C03065	67701	HMDB13222	1.00	1.00	1.00
		erythritol	C00503	222285	HMDB02994	1.59	1.18	0.75
		carotene diol (1)				0.76	0.38	0.49
	Drug	penicillin G	C05551	5904	HMDB15186	1.23	2.61	2.12
		salicylate	C00805	338	HMDB01895	1.01	1.16	1.14
	Chemical	sulfate	C00059	1118	HMDB01448	1.61	3.37	2.09
		HEPES		23831		1.09	1.77	1.62
		phenol red	C12600	4766		1.58	2.94	1.86
		trizma acetate	C07182	6503		1.79	2.03	1.13
		4-acetamidobenzoate	D03836	19266		1.30	2.16	1.66
		thioproline		93176		1.24	2.41	1.94

Table S2: List of biochemical components detected in bovine cumulus-oocyte-complex-conditioned medium.

Bovine COC conditioned maturation media

						Fold of Change		
**Super Pathway**	**Sub Pathway**	**Biochemical Name**	**KEGG**	**PUBCHEM**	**HMDB**	**8h vs. 0h**	**23h vs. 0h**	**23h vs. 8h**
Amino Acid	Glycine, Serine and Threonine Metabolism	glycine	C00037	750	HMDB00123	1.05	1.19	1.13
		N-acetylglycine		10972	HMDB00532	1.06	2.43	2.30
		betaine	C00719	247	HMDB00043	6.80	5.54	0.81
		serine	C00065	5951	HMDB00187	0.79	0.49	0.61
		N-acetylserine		65249	HMDB02931	1.68	4.66	2.78
		threonine	C00188	6288	HMDB00167	1.00	1.08	1.08
		N-acetylthreonine		152204		1.00	2.75	2.75
	Alanine and Aspartate Metabolism	alanine	C00041	5950	HMDB00161	0.98	1.33	1.35
		N-acetylalanine	C02847	88064	HMDB00766	1.87	6.66	3.56
		aspartate	C00049	5960	HMDB00191	0.97	1.04	1.07
		N-acetylaspartate (NAA)	C01042	65065	HMDB00812	1.10	3.21	2.91
		asparagine	C00152	6267	HMDB00168	4.91	54.73	11.16
		N-acetylasparagine		99715	HMDB06028	0.78	0.80	1.03
	Glutamate Metabolism	glutamate	C00025	611	HMDB00148	0.99	1.12	1.13
		glutamine	C00064	5961	HMDB00641	1.02	0.78	0.76
		N-acetylglutamate	C00624	70914	HMDB01138	2.92	7.10	2.43
		N-acetylglutamine	C02716	182230	HMDB06029	4.14	6.02	1.46
		pyroglutamine*		134508		0.42	0.56	1.35
		S-1-pyrroline-5-carboxylate	C04322	1196	HMDB01301	0.68	0.79	1.17
	Histidine Metabolism	histidine	C00135	6274	HMDB00177	0.99	0.93	0.94
		4-imidazoleacetate	C02835	96215	HMDB02024	30.46	51.24	1.68
	Lysine Metabolism	lysine	C00047	5962	HMDB00182	1.09	1.15	1.06
		N6-acetyllysine	C02727	92832	HMDB00206	1.16	4.96	4.28
		N6,N6,N6-trimethyllysine	C03793	440120	HMDB01325	1.06	8.02	7.59
	Phenylalanine Metabolism	phenylalanine	C00079	6140	HMDB00159	1.02	1.07	1.05
		N-acetylphenylalanine	C03519	74839	HMDB00512	1.17	2.16	1.85
		phenylpyruvate	C00166	997	HMDB00205	1.13	1.97	1.74
	Tyrosine Metabolism	tyrosine	C00082	6057	HMDB00158	1.04	1.06	1.03
		4-hydroxyphenylpyruvate	C01179	979	HMDB00707	2.62	2.90	1.11
		3-(4-hydroxyphenyl)lactate	C03672	9378	HMDB00755	1.18	2.74	2.32
		phenol sulfate	C02180	74426	HMDB60015	110.54	204.81	1.85
		o-Tyrosine		91482	HMDB06050	1.00	2.43	2.43
		N-formylphenylalanine		759256		0.81	0.99	1.22
	Tryptophan Metabolism	tryptophan	C00078	6305	HMDB00929	1.04	1.11	1.06
		kynurenine	C00328	161166	HMDB00684	1.47	1.73	1.17
	Leucine, Isoleucine and Valine Metabolism	leucine	C00123	6106	HMDB00687	1.02	1.05	1.02
		N-acetylleucine	C02710	70912	HMDB11756	1.11	2.12	1.91
		4-methyl-2-oxopentanoate	C00233	70	HMDB00695	7.55	17.24	2.28
		isovalerylglycine		546304	HMDB00678	1.16	1.14	0.98
		beta-hydroxyisovalerate		69362	HMDB00754	1.22	1.68	1.38
		isoleucine	C00407	6306	HMDB00172	1.00	1.00	1.00
		3-methyl-2-oxovalerate	C00671	47	HMDB03736	4.92	13.10	2.66
		alpha-hydroxyisovalerate		99823	HMDB00407	1.04	2.02	1.95
		ethylmalonate		11756	HMDB00622	1.13	3.74	3.31
		valine	C00183	6287	HMDB00883	1.01	1.07	1.06
		N-acetylvaline		66789	HMDB11757	1.27	1.81	1.43
		3-methyl-2-oxobutyrate	C00141	49	HMDB00019	1.69	5.63	3.33
		3-hydroxyisobutyrate	C06001	87	HMDB00336	1.08	6.04	5.58
	Methionine, Cysteine, SAM and Taurine Metabolism	methionine	C00073	6137	HMDB00696	1.06	1.10	1.04
		N-acetylmethionine	C02712	448580	HMDB11745	2.77	14.65	5.28
		methionine sulfoxide	C02989	158980	HMDB02005	0.45	0.48	1.06
		cysteine	C00097	5862	HMDB00574	1.84	3.53	1.93
		cysteine s-sulfate	C05824	115015	HMDB00731	0.81	0.52	0.64
		cystine	C00491	67678	HMDB00192	0.82	0.31	0.38
		cysteine sulfinic acid	C00606	109	HMDB00996	1.11	0.81	0.73
		hypotaurine	C00519	107812	HMDB00965	35.65	90.40	2.54
		taurine	C00245	1123	HMDB00251	1.75	1.47	0.84
		3-sulfo-L-alanine	C00506	72886	HMDB02757	0.67	0.58	0.86
	Urea cycle; Arginine and Proline Metabolism	arginine	C00062	232	HMDB00517	1.02	1.02	1.00
		urea	C00086	1176	HMDB00294	1.02	9.68	9.51
		ornithine	C00077	6262	HMDB03374	13.39	38.58	2.88
		2-oxoarginine*	C03771	558	HMDB04225	1.81	2.51	1.38
		proline	C00148	145742	HMDB00162	1.04	1.12	1.08
		dimethylarginine (SDMA + ADMA)	C03626	123831	HMDB01539	6.92	25.13	3.63
		N-acetylproline		322640		0.79	1.52	1.93
		trans-4-hydroxyproline	C01157	5810	HMDB00725	0.98	1.03	1.05
	Creatine Metabolism	guanidinoacetate	C00581	763	HMDB00128	1.18	3.74	3.16
		creatine	C00300	586	HMDB00064	427.32	515.94	1.21
		creatinine	C00791	588	HMDB00562	21.62	20.61	0.95
	Polyamine Metabolism	5-methylthioadenosine (MTA)	C00170	439176	HMDB01173	13.31	3.58	0.27
		N-acetylputrescine	C02714	122356	HMDB02064	1.06	1.49	1.41
	Guanidino and Acetamido Metabolism	4-guanidinobutanoate	C01035	500	HMDB03464	0.73	1.35	1.86
	Glutathione Metabolism	cysteine-glutathione disulfide		4247235	HMDB00656	1.32	3.30	2.50
		5-oxoproline	C01879	7405	HMDB00267	0.77	0.87	1.13
		2-hydroxybutyrate/2-hydroxyisobutyrate				1.84	3.94	2.15
Peptide	Gamma-glutamyl Amino Acid	gamma-glutamylglutamine	C05283	150914	HMDB11738	1.19	1.40	1.17
		gamma-glutamylisoleucine*		14253342	HMDB11170	0.90	1.55	1.72
		gamma-glutamylleucine		151023	HMDB11171	1.00	1.00	1.00
		gamma-glutamylvaline		7015683	HMDB11172	0.88	1.01	1.15
	Acetylated Peptides	phenylacetylglycine	C05598	68144	HMDB00821	0.77	0.86	1.12
Carbohydrate	Glycolysis, Gluconeogenesis, and Pyruvate Metabolism	glucose	C00031	79025	HMDB00122	0.59	0.10	0.17
		pyruvate	C00022	1060	HMDB00243	1.15	3.68	3.19
		lactate	C00186	612	HMDB00190	667.34	1227.15	1.84
		glycerate	C00258	752	HMDB00139	1.43	1.98	1.38
	Pentose Metabolism	ribose	C00121	5779	HMDB00283	0.78	0.85	1.09
		ribitol	C00474	6912	HMDB00508	4.08	6.95	1.70
		ribonate	C01685	5460677	HMDB00867	2.40	2.58	1.07
		arabitol/xylitol	C01904	6912		1.65	3.35	2.02
		arabonate/xylonate				0.84	0.97	1.16
	Glycogen Metabolism	maltose	C00208	10991489	HMDB00163	1.23	1.20	0.97
	Fructose, Mannose and Galactose Metabolism	fructose	C00095	5984	HMDB00660	0.52	0.54	1.04
		mannitol/sorbitol	C00794	5780	HMDB00247	5.90	8.18	1.39
		mannose	C00159	18950	HMDB00169	1.03	0.77	0.75
	Aminosugar Metabolism	erythronate*		2781043	HMDB00613	1.22	2.39	1.95
		N-acetylglucosamine/N-acetylgalactosamine		24139	HMDB00215	1.00	4.52	4.52
Energy	TCA Cycle	citrate	C00158	311	HMDB00094	3.01	5.43	1.80
		aconitate [cis or trans]				1.00	1.96	1.96
		alpha-ketoglutarate	C00026	51	HMDB00208	11.53	21.69	1.88
		succinate	C00042	1110	HMDB00254	3.66	5.44	1.49

		fumarate	C00122	444972	HMDB00134	1.58	4.59	2.90
		malate	C00149	525	HMDB00156	1.70	7.08	4.17
	Oxidative Phosphorylation	phosphate	C00009	1061	HMDB01429	1.05	1.06	1.01
Lipid	Medium Chain Fatty Acid	caproate (6:0)	C01585	8892	HMDB00535	1.00	1.57	1.57
		heptanoate (7:0)	C17714	8094	HMDB00666	0.64	0.49	0.77
		caprylate (8:0)	C06423	379	HMDB00482	1.00	1.00	1.00
	Long Chain Fatty Acid	erucate (22:1n9)	C08316	5281116	HMDB02068	1.00	1.00	1.00
	Polyunsaturated Fatty Acid (n3 and n6)	linolenate [alpha or gamma; (18:3n3 or 6)]	C06426	5280934	HMDB03073	1.85	0.99	0.54
	Fatty Acid, Dicarboxylate	glutarate (pentanedioate)	C00489	743	HMDB00661	0.68	1.23	1.81
		2-hydroxyglutarate	C02630	43	HMDB00606	1.78	3.23	1.82
		maleate	C01384	444266	HMDB00176	1.05	1.37	1.30
		azelate (nonanedioate)	C08261	2266	HMDB00784	0.95	1.29	1.37
	Carnitine Metabolism	carnitine	C00318	10917	HMDB00062	1.77	1.57	0.89
	Fatty Acid, Monohydroxy	3-hydroxyhexanoate		151492		1.00	1.05	1.05
		3-hydroxyoctanoate		26613	HMDB01954	1.00	1.00	1.00
	Inositol Metabolism	myo-inositol	C00137	892	HMDB00211	11.70	6.74	0.58
	Phospholipid Metabolism	choline	C00114	305	HMDB00097	0.36	0.31	0.88
		choline phosphate	C00588	1014	HMDB01565	75.06	105.31	1.40
		glycerophosphorylcholine (GPC)	C00670	71920	HMDB00086	1.21	1.88	1.56
		glycerophosphoethanolamine	C01233	123874	HMDB00114	1.21	1.97	1.62
		glycerophosphoinositol*		167572		1.27	3.17	2.49
	Phosphatidylcholine (PC)	1-palmitoyl-2-oleoyl-GPC (16:0/18:1)		6436017	HMDB07972	2.29	2.59	1.13
	Glycerolipid Metabolism	glycerol	C00116	753	HMDB00131	6.89	10.13	1.47
		glycerophosphoglycerol	C03274	439964		4.13	10.12	2.45
	Mevalonate Metabolism	3-hydroxy-3-methylglutarate	C03761	1662	HMDB00355	4.22	17.81	4.22
Nucleotide	Purine Metabolism, (Hypo)Xanthine/Inosine containing	inosine	C00294	6021	HMDB00195	451.95	119.45	0.26
		hypoxanthine	C00262	790	HMDB00157	6.93	13.06	1.88
		xanthine	C00385	1188	HMDB00292	1.46	2.36	1.62
		2'-deoxyinosine	C05512	65058	HMDB00071	1.45	1.11	0.77
		allantoin	C02350	204	HMDB00462	1.20	1.09	0.91
	Purine Metabolism, Adenine containing	adenosine-2',3'-cyclic monophosphate	C02353	2024	HMDB11616	1.73	1.11	0.64
		adenosine	C00212	60961	HMDB00050	10.85	1.54	0.14
		adenine	C00147	190	HMDB00034	0.51	0.08	0.16
		2'-deoxyadenosine	C00559	13730	HMDB00101	0.83	0.91	1.10
	Purine Metabolism, Guanine containing	guanosine	C00387	6802	HMDB00133	2.16	1.26	0.58
		guanine	C00242	764	HMDB00132	0.73	1.83	2.51
		7-methylguanine	C02242	11361	HMDB00897	1.37	4.27	3.11
	Pyrimidine Metabolism, Orotate containing	orotate	C00295	967	HMDB00226	6.85	35.24	5.15
	Pyrimidine Metabolism, Uracil containing	uridine	C00299	6029	HMDB00296	24.46	98.06	4.01
		uracil	C00106	1174	HMDB00300	1.02	0.88	0.87
		pseudouridine	C02067	15047	HMDB00767	6.31	22.58	3.58
		2'-deoxyuridine	C00526	13712	HMDB00012	13.24	32.33	2.44
		3-ureidopropionate	C02642	111	HMDB00026	1.17	1.64	1.40
	Pyrimidine Metabolism, Cytidine containing	cytidine	C00475	6175	HMDB00089	3.29	16.52	5.02
		2'-deoxycytidine	C00881	13711	HMDB00014	13.33	33.91	2.54
	Pyrimidine Metabolism, Thymine containing	thymidine	C00214	5789	HMDB00273	3.36	6.30	1.88
		thymine	C00178	1135	HMDB00262	0.63	0.40	0.63
		5,6-dihydrothymine	C00906	93556	HMDB00079	1.26	2.27	1.80
Cofactors and Vitamins	Nicotinate and Nicotinamide Metabolism	nicotinate	C00253	938	HMDB01488	1.09	1.10	1.01
		nicotinamide	C00153	936	HMDB01406	0.29	0.59	2.08
	Pantothenate and CoA Metabolism	pantothenate	C00864	6613	HMDB00210	1.10	0.60	0.54
	Ascorbate and Aldarate Metabolism	threonate	C01620	151152	HMDB00943	12.67	12.20	0.96
		gulonate*	C00257	9794176	HMDB03290	3.67	6.36	1.73
	Tocopherol Metabolism	alpha-tocopherol	C02477	14985	HMDB01893	1.84	2.04	1.11
	Biotin Metabolism	biotin	C00120	171548	HMDB00030	1.06	1.59	1.50
	Vitamin B6 Metabolism	pyridoxine (Vitamin B6)	C00314	1054	HMDB02075	0.75	0.47	0.63
		pyridoxamine	C00534	1052	HMDB01431	0.90	0.84	0.94
		pyridoxal	C00250	1050	HMDB01545	2.76	5.38	1.95
		pyridoxate	C00847	6723	HMDB00017	0.98	1.95	2.00
Xenobiotics	Benzoate Metabolism	hippurate	C01586	464	HMDB00714	1.72	1.29	0.75
		benzoate	C00180	243	HMDB01870	0.69	0.83	1.20
		p-cresol sulfate		4615423	HMDB11635	2.21	2.18	0.99
	Food Component/Plant	maltol	C11918	8369	HMDB30776	0.29	0.64	2.19
		gluconate	C00257	10690	HMDB00625	0.83	1.07	1.29
		erythritol	C00503	222285	HMDB02994	13.06	22.90	1.75
		stachydrine	C10172	115244	HMDB04827	1.00	1.00	1.00
	Drug	streptomycin		5999		0.99	0.99	1.00
		penicillin G	C05551	5904	HMDB15186	1.93	6.67	3.46
		salicylate	C00805	338	HMDB01895	1.26	2.39	1.90
	Chemical	sulfate*	C00059	1118	HMDB01448	1.05	1.09	1.04
		HEPES		23831		7870.60	11746.25	1.49
		phenol red	C12600	4766		0.98	0.99	1.01
		4-acetamidobenzoate	D03836	19266		1.08	1.53	1.42
		thioproline		93176		3.38	7.26	2.15
